# Mathematical Modeling of Human Retinal Vascular Pattern Around the Foveal Avascular Zone

**DOI:** 10.1167/tvst.15.3.1

**Published:** 2026-03-02

**Authors:** Kotaro Yoshimura, Kei Sugihara, Ichiro Maruko, Tomohiro Iida, Takashi Miura

**Affiliations:** 1 Department of Anatomy and Cell Biology, Kyushu University Graduate School of Medical Sciences, Fukuoka, Japan; 2 Department of Ophthalmology, Tokyo Women's Medical University, Tokyo, Japan; 3 Department of Ophthalmology, Ageo Central General Hospital, Ageo, Japan

**Keywords:** retinal development, retinal vasculature, mathematical modeling, fovea, retinal astrocytes

## Abstract

**Purpose:**

Human retinas have unique anatomical structures called the macula and an associated characteristic vascular pattern. Despite its clinical importance, the mechanism underlying the human-specific vascular pattern remains unknown because of limitations in experimental approaches using primate samples. This study aimed to elucidate such vascular formation process.

**Methods:**

We first examined the effects of four hypothetical factors contributing to foveal avascular zone (FAZ) formation: inhibitory molecule secretion, chemoattractant depletion, tissue deformation (towing), and tip cell migration restriction. None reproduced the features of the human retinal vascular structure. Next, we developed a mathematical model of human retinal vascular development by considering endothelial cells and astrocytes. We assumed that retinal vessels form via angiogenesis according to the gradient of vascular endothelial growth factor and that astrocytes dynamically expand while avoiding the fovea, providing scaffolds for angiogenesis.

**Results:**

Our astrocyte-coupling model recapitulated various features of the human retinal vascular pattern, including a radially outward vascular pattern from the optic disc, inferior and superior temporal arcades, FAZ formation, a radially inward vascular pattern around FAZ, and a vertically facing pattern toward the horizontal vascular borderline in the temporal region of FAZ. Other models did not support the other four hypotheses.

**Conclusions:**

Our model explained human-specific retinal vascular formation as the combination of angiogenesis and vascular growth restriction by retinal astrocytes. These results also suggest the importance of astrocyte dynamics, particularly their timing of spreading.

**Translational Relevance:**

Our modeling framework can be extended to abnormal vascular patterns observed in diseases, including retinopathy of prematurity.

## Introduction

**Figure 1.**
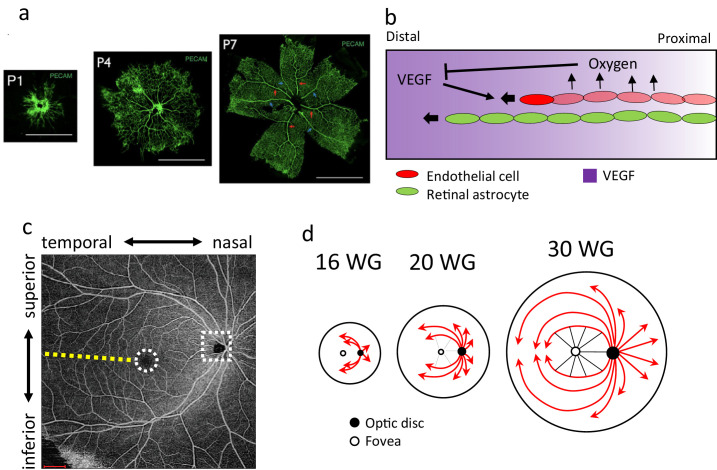
Retinal development in mouse and human. (**a**) Mouse retina vasculature at different neonatal stages (modified from^70^). (**b**) The schematic image of the retinal developmental process. (**c**) OCTA image of human retinal vasculature. *Yellow dotted line*: the horizontal vascular boundary in the temporal region of FAZ. *White dotted circle:* FAZ. *White dotted rectangle*: OD. (**d**) The schematic image of the growth pattern of human retinal vasculature with a focus on the fovea (redrawn from^16^). *Scale bars*, 1 mm.

Retinal vascular development has been extensively studied, and key factors have been elucidated using a mouse model^1^^,^^2^ (Figs. 1a, 1b). During development, astrocytes first invade and extend in the retina from the optic disc, forming a network structure and covering the inner surface of the retina.^3^ Next, endothelial cells enter the retina, proliferate, and migrate from the optic disc to the periphery according to the gradient of vascular endothelial growth factor (VEGF).^4^^–^^7^ Astrocytes support endothelial tip cell migration for angiogenesis through the secretion of VEGF,^4^^,^^5^^,^^8^ supply of fibronectin as matrix scaffolds,^9^^–^^11^ and cell-cell adhesions between astrocytes and the endothelial filopodia.^12^

Adult human retina structures have several different characteristics compared with mice. Human retinas have the macula, the structure that enables high acuity at its center, including the fovea, the central pit in the macula. Cone cells are centralized in the human macula, which contributes to color vision and high acuity. Moreover, the fovea is believed to contribute to high visual acuity by magnifying the image.^13^ In fact, patients with foveal hypoplasia, a congenital failure of foveal development, have visual impairment.^14^ Despite such importance, the mechanisms of the fovea formation are not well-understood owing to a lack of good experimental models.

Retinal vascular developmental processes and the vascular patterns are different between mice (Fig. 1a) and humans (Fig. 1c). In mouse retinal vascular development, the immature vascular plexus is morphed into hierarchic vascular structures by blood flow-driven remodeling processes.^2^ In contrast, large vessels are radially formed before capillaries fill the avascular space in human retinal development.^15^ These differences indicate that the human retinal vascular development process, to which angiogenesis may contribute,^16^ differed from that of the mouse. The human retinal vascular network has a structure in which endothelial cells spread from the optic disc at the nasal side, avoiding the fovea, and form a radially inward vascular pattern toward its center (Fig. 1d). A region without vasculature around the fovea is called the foveal avascular zone (FAZ) (Fig. 1c, dotted white circle), which does not exist in the murine reitna.^17^ These vascular features are also observed in other primates, which have the fovea and FAZ.^18^^,^^19^ The previous study showed that FAZ was formed by vascular formation avoiding it, not by leaving vessels on the fovea,^20^ including pruning, destruction, or removal of vessels. Astrocytes also do not distribute around the fovea.^21^ To our knowledge, the FAZ formation mechanism is unknown.

Optical coherence tomography angiography (OCTA) is a recently advanced noninvasive method for ocular vasculature imaging using OCT. OCT measures the amplitude and delay of reflected or backscattered light (interferometry).^22^ We can calculate differences among the scans by comparing repeated OCT scans to obtain the region with blood flow because the reflectivity and scattering changes from one scan to the next only in such regions.^22^ The spatial resolution of OCTA is about 10 µm, and this method can capture three-dimensional structures of retinal capillaries, which conventional fluorescein angiography cannot.

Various mathematical models have been proposed for vascular development, but no models have been developed for human retinal vascular development. A classical model is the Chaplain-Anderson model,^23^ which was designed for tumor angiogenesis. In this model, tip cells, which are leading endothelial cells, are assumed to migrate according to random walk, chemotaxis, and haptotaxis, and other endothelial cells are assumed to follow tip cells. In addition, several other studies reproduced various vascular patterns by assuming tip cell migrations controlled by random walk, chemotaxis, and other factors, such as haptotaxis, interactions between chemotactic factors and their associated proteases, or vessel regression.^24^^–^^29^ Some of them incorporated the uptake of chemoattractants by vessels following tip cell migration, which affected tip cell migration in other branches.^23^^,^^26^^–^^29^ These models have been widely studied in mathematical biology because they are relatively easy to analyze mathematically. Additionally, this model was applied to mouse retinal development in conjunction with various other factors.^30^ Another model type is L-system-like model.^31^^,^^32^ The L-system model is a rule-based model in which the growth and branching of vessels are implemented according to a certain set of rules. Starting from initial segments, the rule-based operations are repeated, resulting in a tree-like structure. Other previous studies reproduced a hierarchical vascular network in the mouse retina using a hybrid partial differential equation-discrete modeling approach, taking into account blood flow-driven vascular remodeling.^30^ Furthermore, a mathematical model that integrates angiogenesis and vascular remodeling while incorporating astrocyte effects has been applied to murine retinal vascular development.^33^ However, these models do not describe human retinal vasculature development. Especially, the mechanism of human-specific vascular pattern formation has never been explored in modeling studies.

In this study, we constructed a mathematical model of human retinal vascular development with tip cell migrations regulated by random walk and chemotaxis. Specifically, we considered astrocyte distribution in the model and successfully reproduced human-like vascular patterns, especially around the FAZ. Our results also indicate that astrocyte dynamics play a crucial role in the development of human-specific vascular patterns around the fovea.

## Materials and Methods

### Mathematical Model

#### Endothelial Cell Dynamics

First, for considering the human retinal vascular model, we assumed that vessels grow in a two-dimensional plane because we supplementarily checked that the fact that the eyes were spherical was not the cause of the human retinal vascular features (Supplementary Fig. S1, Supporting information–Assessment of OCTA image distortion caused by the projection of spherical surface into the plane). We defined the location of *i*-th tip cell at time *t* $\vec{r}\left(i,t\right)=\left(r_{x}\left(i,t\right),r_{y}\left(i,t\right)\right)$ in continuous space, which moved against the gradient of oxygen concentration *u*(*x*, *y*, *t*). Initially, the tip cells were located near the optic disc, and they moved according to the following rule:

(1) $$\begin{array}{ccc} \vec{r}\left(i,t+\Delta t\right) & = & \vec{r}\left(i,t\right)\;+\Delta t\xi \left(x,y,t\right)\gamma \left(x,y,t\right)\\ & & \times \left(\alpha \vec{\eta }\left(i,t\right)-\beta \nabla u\left(x,y,t\right)\right). \end{array}$$

α and β were the strengths of random walk and chemotaxis, respectively. Δ*t* is the unit time of temporal discretization. $\vec{\eta }\left(i,t\right)=\left(\cos(\theta (i,t)),\sin(\theta (i,t))\right)$ represented the random cell migration. θ(*i*, *t*) was determined from a uniform distribution between 0 and 2π for each tip cell at every time step. We defined the tip cells as moving at a constant speed in a random direction.

We calculated other factors except for tip cells in a discrete space of an Δ*x* × Δ*x* grid. Then, we rounded the tip cell coordinates (*r*_*x*_, *r*_*y*_) to obtain (*x*, *y*), which were used for the subsequent calculations.

(2) $$\nabla u\left(x,y,t\right)=\left(\frac{\partial }{\partial x}u\left(x,y,t\right),\frac{\partial }{\partial y}u\left(x,y,t\right)\right)$$

was a concentration gradient of oxygen. We assumed that VEGF was a chemoattractant of tip cells, demonstrated in the murine retina.^7^ In our model, we used oxygen gradients instead of VEGF for tip cell chemotaxis because VEGF was highly expressed in hypoxic regions.^4^^–^^6^ A previous study showed that excessive VEGF can inhibit human umbilical vein endothelial cell migration.^34^ However, in our model, we assumed that tip cell migration did not slow down under high VEGF concentrations, which corresponded with hypoxia, because VEGF concentration in the normal developmental retina was not expected to reach the excessive levels observed in vitro.

Assuming oxygen supplies from retinal vessels and oxygen decomposition proportional to oxygen, we defined oxygen distribution *k*_oxygen_ from a point source as the solution of the following reaction-diffusion equation:

(3) $$0=-q_{\text{oxygen}}u+D_{\text{oxygen}}\nabla^{2}u,$$

(4) $$u\left(x,y,t\right)=u_{0}\;\mathrm{on}\;l\left(x_{0},y_{0},x,y\right)=l_{\text{vessel}},$$

where *q*_oxygen_ is the consumption rate, *D*_oxygen_ is the diffusion coefficient, *l*_vessel_ is the vessel diameter, and $l\left(x_{0},y_{0},x,y\right)=\sqrt{{\left(x-x_{0}\right)}^{2}+{\left(y-y_{0}\right)}^{2}}$. The solution is as follows:

(5) $$\begin{array}{c} \begin{array}{ccc} & & \;k_{\mathrm{oxygen}}\left(x_{0},y_{0},x,y\right)\\ & & \;=\left\{\begin{array}{cc} u_{0} & l\left(x_{0},y_{0},x,y\right)<l_{\text{vessel}},\\ \frac{u_{0}\left(J_{0}\left(i\sqrt{\frac{q_{\text{oxygen}}}{D_{\text{oxygen}}}}l\left(x_{0},y_{0},x,y\right)\right)-iY_{0}\left(-i\sqrt{\frac{q_{\text{oxygen}}}{D_{\text{oxygen}}}}l\left(x_{0},y_{0},x,y\right)\right)\right)}{J_{0}\left(i\sqrt{\frac{q_{\text{oxygen}}}{D_{\text{oxygen}}}}l_{\text{vessel}}\right)-iY_{0}\left(-i\sqrt{\frac{q_{\text{oxygen}}}{D_{\text{oxygen}}}}l_{\text{vessel}}\right)} & l\left(x_{0},y_{0},x,y\right)\geq l_{\text{vessel}}. \end{array}\right. \end{array} \end{array}$$

Where *J*_0_ and *Y*_0_ are zeroth-order Bessel functions of the first and second kinds, respectively. The distribution of oxygen was determined by the sum of the oxygen distribution from point sources as follows:

(6) $$u\left(x,y,t\right)=\sum_{t}\sum_{i}k_{\mathrm{oxygen}}\left(r_{x}\left(i,t\right),r_{y}\left(i,t\right),x,y\right).$$

We also assumed that branching occurred only at tip cell positions, following the previous study^23^ with minor modifications, and that hypoxia induced branching. A tip cell $\vec{r}\left(i,t\right)$ divided into two cells $\vec{r}\left(i,t\right)$ and $\vec{r}\left(N\left(t\right)+1,t\right)$ at probability *p*_branch_Δ*t* when *u*(*r*_*x*_(*i*, *t*), *r*_*y*_(*i*, *t*)) < *u*_branch_, where *N*(*t*) was the number of tip cell at time *t*. In the previous study,^23^ two new tip cells emerge at the same position when a vessel branches, which causes the unrealistic situation where these two cells move along the same route for a while. To solve this problem, we defined the positions of new tip cells according to the following rule:

(7) $$\vec{r}\left(i,t+\Delta t\right)=\vec{r}\left(i,t\right)+b\left(\theta_{0},\theta_{\mathrm{br}},\phi \right),\;$$

(8) $$\vec{r}\left(N\left(t\right)+1,t+\Delta t\right)=\vec{r}\left(i,t\right)+b\left(\theta_{0},-\theta_{\mathrm{br}},\phi \right),\;$$

(9) $$\begin{array}{ccc} b(\theta_{0},\theta_{\mathrm{br}},\phi ) & = & \left(\cos\left(\theta_{0}+\theta_{\mathrm{br}}/2+\phi \right),\right.\\ & & \left.\sin\left(\theta_{0}+\theta_{\mathrm{br}}/2+\phi \right)\right),\; \end{array}$$

where θ_0_ is the direction of the parent branch, θ_br_ is the fixed angle between two new branches, and ϕ is the angle between the extension of the straight line of the parent branch and the line connecting the branching point and the midpoint of the new tip cells. We determined ϕ from a uniform distribution between −(π − θ_br_)/2 and (π − θ_br_)/2. Our branching rule allows the two new tip cells to migrate in different directions from the existing vascular structure. Furthermore, this mechanism stochastically enables lateral branching if one daughter branch continues in the same direction as the parent vessel and the other deviates at an angle.

We also assumed that tip cell speed decreased exponentially when the local oxygen concentration at the tip cell was larger than *u*_branch_:

(10) $$\xi \left(\vec{r}\right)=\left\{\begin{array}{cc} 1 & u\left(x,y,t\right)<u_{\text{branch}},\\ \exp\left(-\frac{u\left(x,y,t\right)-u_{\text{branch}}}{k_{\xi }}\right) & u\left(x,y,t\right)\geq u_{\text{branch}}, \end{array}\right.$$

which resembles how substances deposited along individual cellular pathways modulate subsequent cell motility in a previous mathematical model.^35^ This term was added to prevent tip cells from continuing to migrate when they were surrounded by vascular structures because there was a certain interval between the sufficiently thick vessels on which we focused in OCTA images. This prevented some tip cell trajectories from overlapping.

We assumed that vessels can grow only in the region where astrocytes already exist. To incorporate the effect of astrocytes on vascular development, we modified the cell migration rate with γ(*x*, *y*, *t*) (see Mathematical Model–Astrocyte Dynamics for the detailed description).

*N*_init_ tip cells were initially placed at distance ρ_init_ from the optic disc. To prevent tip cells from approaching the optic disc, we assumed oxygen supply *u*_OD_(*x*, *y*) from the optic disc:

(11) $$\begin{array}{ccc} & & \;u_{\mathrm{OD}}\left(x,y\right)\\ & & \;=\;u_{\mathrm{Threshold}}\;\exp\;\left(\;\;-\;\;{\left(\;\frac{\sqrt{{\left(x\;-\;x_{\mathrm{OD}}\right)}^{2}\;+\;{\left(y\;-\;y_{\mathrm{OD}}\right)}^{2}}}{\rho_{\text{init}}}\right)}^{\;\;2}\right)\;\;, \end{array}$$

where (*x*_OD_, *y*_OD_) = (*d*_OD_, 0) is the position of the optic disc center. *u*_OD_(*x*, *y*) corresponds with oxygen supplies from blood vessels on the optic disc, including the central retinal artery, which our model did not explicitly reproduce.

#### Astrocyte Dynamics

We assumed that the growth speed *V*(*C*_inhibitor_(*x*, *y*)) of the astrocyte distribution γ(*x*, *y*, *t*) would be approximately as follows:

(12) $$\begin{array}{ccc} & & \;V(C_{\mathrm{inhibitor}}\left(x,y\right))\\ & & ∼\max(0,v_{\text{astrocyte}}\left(1-k_{i}C_{\mathrm{inhibitor}}\left(x,y\right)\right)), \end{array}$$

where *C*_inhibitor_(*x*, *y*) is the concentration of the astrocyte inhibitor, *k*_*i*_ is the coefficient of the inhibitory effects, and *v*_astrocyte_ is the spreading velocity in the absence of the inhibitor.

According to this assumption, we constructed an astrocyte dynamics model based on the Eden growth model.^36^ In our model, astrocyte expansion is controlled by the spreading probability P(C inhibitor (x,y))=ΔtΔxV(C inhibitor (x,y)). At a lattice (*x*, *y*) where γ(*x*, *y*, *t*) = 0, we set γ(*x*, *y*, *t* + Δ*t*) = 1 with the probability *P*(*C*_inhibitor_(*x*, *y*)) if γ = 1 for any adjacent lattice to (*x*, *y*); otherwise, γ(*x*, *y*, *t* + Δ*t*) = 0. If γ(*x*, *y*, *t*) = 1, always γ(*x*, *y*, *t* + Δ*t*) = 1.

Under this process, astrocyte expansion stops at a lattice (*x*, *y*) where *P*(*C*_inhibitor_(*x*, *y*)) = 0. To satisfy this requirement in *d*_fovea_(*x*, *y*) ⩽ ρ_FAZ_, we defined *C*_inhibitor_(*x*, *y*) as follows:

(13) $$\begin{array}{ccc} & & \;C_{\mathrm{inhibitor}}\left(x,y\right)\\ & & \;=\exp\;\left(\;\frac{1}{{\left(\frac{d_{\text{inhibitor}}}{\rho_{\text{FAZ}}}\right)}^{2}-1}\left(\;1-{\left(\frac{d_{\mathrm{fovea}}\left(x,y\right)}{\rho_{\text{FAZ}}}\right)}^{2}\right)\;\right)\;, \end{array}$$

where *d*_inhibitor_ is the controlling parameter of the inhibitor diffusion length, and *d*_fovea_(*x*, *y*) is the distance from the foveal center, d fovea (x,y)=x-x fovea 2+y-y fovea 2. In addition, we excluded the death of astrocytes from our model for simplification because astrocytes did not die often around the FAZ,^37^ which indicated that astrocyte death did not relate to FAZ formation.

At *t* = 0, astrocytes distribute within a radius *r*_astrocyte_ from the optic disc.

In the no-astrocyte dynamics model, we used the fixed astrocyte distribution γ_fixed_(*x*, *y*), which was the final distribution of the astrocyte dynamics model. Therefore, we calculated tip cell migration in this model as follows:

(14) $$\begin{array}{ccc} \vec{r}\left(i,t+\Delta t\right) & = & \vec{r}\left(i,t\right)+\Delta t\xi \left(x,y,t\right)\gamma_{\text{fixed}}\left(x,y\right)\\ & & \times \left(\alpha \vec{\eta }\left(i,t\right)-\beta \nabla u\left(x,y,t\right)\right). \end{array}$$

#### FAZ Formation Hypotheses

To assess whether mechanisms other than the astrocyte effect are capable of forming the vascular pattern around FAZ, we also tested four hypotheses that may reproduce the vascular pattern. We modified the following equation:

(15) $$\begin{array}{ccc} \vec{r}\left(i,t+\Delta t\right) & = & \vec{r}\left(i,t\right)\;+\Delta t\xi \left(x,y,t\right)\\ & & \times \left(\alpha \vec{\eta }\left(i,t\right)-\beta \nabla u\left(x,y,t\right)\right). \end{array}$$

In inhibitory molecule hypothesis, we assumed an endothelial inhibitor distribution in the fovea *C*_molecule_(*x*, *y*) is:

(16) $$\begin{array}{ccc} & & \;C_{\mathrm{molecule}}\left(x,y\right)\\ & & \;=\exp\;\left(\;-\;{\left(\;\frac{\sqrt{{\left(x-x_{\mathrm{fovea}}\right)}^{2}+{\left(y-y_{\mathrm{fovea}}\right)}^{2}}}{\rho_{\text{endo,inh}}}\right)}^{2}\;\right)\;, \end{array}$$

where ρ_endo,inh_ is the diffusion length of the inhibitor, and (*x*_fovea_, *y*_fovea_) = (0, 0) is the position of the foveal center. We applied *C*_molecule_(*x*, *y*) to Equation (15) according to the following rule:

(17) $$\begin{array}{ccc} & & \;\vec{r}\left(i,t+\Delta t\right)\;=\vec{r}\left(i,t\right)\;+\Delta t\xi \left(x,y,t\right)\left(\alpha \vec{\eta }\left(i,t\right)\right.\\ & & \left.-\beta \nabla u\left(x,y,t\right)-\beta_{\mathrm{molecule}}\nabla C_{\mathrm{molecule}}\left(x,y\right)\right), \end{array}$$

where β_molecule_ = 1 is the inhibitory coefficient.

In the no-chemoattractant hypothesis, we considered the distribution of a chemoattractant, such as VEGF *C*_VEGF_:

(18) $$\begin{array}{ccc} & & \;\vec{r}\left(i,t+\Delta t\right)\;=\vec{r}\left(i,t\right)+\Delta t\xi \left(x,y,t\right)\\ & & \times \left(\alpha \vec{\eta }\left(i,t\right)+\beta \nabla C_{\mathrm{VEGF}}\left(x,y,t\right)\right). \end{array}$$

We assumed VEGF is highly expressed in hypoxic regions and downregulated around the fovea by an unknown mechanism:

(19) $$\begin{array}{ccc} & & \;C_{\mathrm{VEGF}}\left(x,y,t\right)=C_{\mathrm{baseline}}-u\left(x,y,t\right)\\ & & \;-C_{\mathrm{baseline}}\exp\;\left(\;-\;{\left(\;\frac{\sqrt{{\left(x\;-\;x_{\mathrm{fovea}}\right)}^{2}\;+\;{\left(y\;-\;y_{\mathrm{fovea}}\right)}^{2}}}{\rho_{\text{VEGF}}}\right)}^{2}\right),\;\;\;\; \end{array}$$

where *C*_baseline_ is the baseline expression level of VEGF. We used ρ_VEGF_ as the VEGF diffusion length.

In the towing hypothesis, we assumed retinal vessels develop uniformly around the fovea and are later pushed away by tissue deformation. To implement this, we initially simulated endothelial dynamics by utilizing Equation (15) (Supplementary Fig. S2a). After that, we radially moved the points at distance 0 ⩽ *d*_fovea_ < 2ρ_FAZ_ from the foveal center to other coordinates at distance d fovea +2ρFAZ2 (Supplementary Fig. S2b).

In the keep-out hypothesis, to prevent tip cells from approaching the fovea, we restricted the area where they can move:

(20) $$\gamma_{\mathrm{fixed}}\left(x,y\right)=\left\{\;\begin{array}{cc} 0 & \sqrt{{\left(x\;-\;x_{\mathrm{fovea}}\right)}^{2}\;+\;{\left(y\;-\;y_{\mathrm{fovea}}\right)}^{2}}\;<\;\rho_{\text{FAZ}},\\ 1 & \text{otherwise}, \end{array}\right.\;$$

(21) $$\begin{array}{ccc} & & \vec{r}\left(i,t+\Delta t\right)=\vec{r}\left(i,t\right)+\Delta t\xi \left(x,y,t\right)\\ & & \;\times \;\gamma_{\mathrm{fixed}}\left(\vec{r}\right)\left(\alpha \vec{\eta }\left(i,t\right)-\beta \nabla u\left(x,y,t\right)\right).\; \end{array}$$

#### Boundary Conditions

We performed calculations of the distribution of diffusive molecules (e.g., *u*(*x*, *y*, *t*)) in a square area with periodic boundary conditions. The expansion of astrocyte distribution was restricted to a circular area with a radius ρ_retina_, corresponding with the retina size. Because astrocytes did not extend beyond this area, tip cell migration was also restricted in this region. Although oxygen and astrocyte inhibitors diffuse outside this circle, even under periodic boundary conditions, their effects on the opposite side of the domain were negligible due to their short diffusive lengths.

#### Numerical Simulation

Numerical simulation of the model was implemented by Python^38^ and Mathematica (Wolfram Research).

#### Parameter Selection

We set each parameter as shown in Supplementary Table S1. We used these parameters in all calculations except for the case of searching parameters, which fit OCTA data or checking the effects of parameters. We determined a lattice length Δ*x* and a discrete time Δ*t* from the distance between FAZ and OD *d*_OD_ = 4 mm^17^ and the retinal developmental time 4200 hour, which corresponded with the whole numerical time. We selected the parameters, α, β, *u*_branch_, *p*_branch_, *v*_astrocyte_, and *d*_inhibitor_, so that our model results match the OCTA data (Supplementary Fig. S3, Supporting Information–Parameter Selection in our model). θ_br_ was chosen to match the apparent branching angle in the numerical simulations with the measured angle in OCTA images (Supplementary Fig. S11c).

We also performed a sensitivity analysis of our model to parameters other than those mentioned above, as discussed in Supporting Information–Parameter Variation. We checked the influences of the ratio governing oxygen distribution, defined as $R_{\text{oxygen}}:=\frac{q_{\text{oxygen}}}{D_{\text{oxygen}}}$ on the vascular density (Supplementary Fig. S11a). We also investigated the changes in vascular density under varying levels of oxygen-dependent migration inhibition (Supplementary Fig. S11b). Furthermore, we revealed that θ_br_ had minimal influence on the actual branching angles (Supplementary Fig. S11c). We additionally analyzed the sensitivity to the initial patterns (Supplementary Fig. S12) and the discrete time step Δ*t* (Supplementary Fig. S13). These sensitivity analyses confirmed that the selected parameters, *R*_oxygen_, *u*_migration_, *k*_ξ_, *N*_init_, and ρ_init_, were appropriate for reproducing the vascular structures similar to those observed in the OCTA data, and also revealed the influence of Δ*t* on the branching process. We note that the model paramters for oxygen and inhibitor concentrations have the dimension of 1/ mm 3, defined as per unit volume.

### OCTA

All eyes were examined by spectral-domain OCTA (RTVue AVANTI, Optovue, Fremont, CA) with a 3 × 3 mm scan centered on the fovea and/or swept-source OCTA (AngioPlex Elite 9000, Zeiss, Jena, Germany) with a 12 × 12 mm scan centered on the fovea. The RTVue XR Avanti, with a light source of approximately 840 nm, can visualize microvascular structures using the split-spectrum amplitude-decorrelation angiography algorithm. This system achieves 70,000 A-scans/second with motion correction technology. The software included with the RTVue XR Avanti automatically provides four OCTA images divided into four depths to show the superficial capillary plexus, deep capillary plexus, outer retina, and choriocapillaris layers. Analysis of the FAZ area was used in each image of the superficial capillary plexus. The AngioPlex Elite 9000, with a light source emission between 1040 and 1060 nm, acquires 100,000 A-scans/second and includes a real-time eye-tracking system (FastTrac). For analysis of the retinal vasculature, even larger areas, including the FAZ, were used in images with segmentation of the entire retinal layers. At the same time, cross-sectional OCT images were obtained with these devices. Eyes with poor image quality were excluded.

### OCTA Image Processing

OCTA images processing were performed by using NumPy,^39^ scipy,^40^ and OpenCV^41^ in Python.^38^

#### Detection of the Optic Disc and the FAZ

To identify the location of the FAZ and the optic disc, OCTA images were sequentially processed with a high-pass filter, smoothing, and binarization. The upper and lower zones, where neither FAZ nor the optic disc is located, were excluded. Avascular regions were detected, excluding the 150-pixel upper and lower ends of each image. The avascular region closest to the image center was identified as FAZ, and the largest avascular region was identified as the optic disc (Supplementary Fig. S4a).

#### OCTA Image Alignment

The original OCTA images were scaled and rotated to align the FAZ and the optic disc horizontally at a fixed distance. Then, each image was cropped into a FAZ-centered square with the side length equal to twice the OD-FAZ distance. Images were also horizontally flipped when necessary (Supplementary Fig. S4b).

#### Vascular Structure Identification

Aligned OCTA images were pre-processed with a high-pass filter. Regions identified as FAZ or optic disc were then removed. Vessel structures were emphasized using the Meijering filter^42^ and then skeletonized (Supplementary Fig. S4c).

#### Extraction of Vascular Branch Coordinates

Skeleton pixels surrounded by three or more neighboring skeleton pixels were regarded as vascular junction regions. To identify individual branches, we removed junction pixels to distinguish each vascular branch segment. Additionally, branch segments that were too small were deleted.

In each segment, two endpoints were identified as the pixels that have only one neighboring branch pixel. Starting from one endpoint, the neighboring pixel was iteratively identified to obtain the sorted list of the pixel coordinates along the branches.

Along a branch, every fifth pixel was selected as a control point *P*_*i*_ (0 ⩽ *i* ⩽ *m* − 1) for B-spline fitting. Given these control points, the B-spline function *S*(*t*) and the basis function *b*_*j*, *k*_(*t*) were expressed as follows:

(22) $$S\left(t\right)=\sum_{i=0}^{m-1}P_{i}b_{i,n}\left(t\right),\;$$

(23) $$b_{j,0}\left(t\right)=\left\{\begin{array}{cc} 1 & t_{j}\leq t<t_{j+1},\\ 0 & \mathrm{otherwise}, \end{array}\right.\;$$

(24) $$\begin{array}{ccc} b_{j,k}\left(t\right) & = & \frac{t-t_{j}}{t_{j+k}-t_{j}}b_{j,k-1}\left(t\right)\\ & & +\frac{t_{j+k+1}-t}{t_{j+k+1}-t_{j+1}}b_{j+1,k-1}\left(t\right),\; \end{array}$$

where the degree of B-spline was set to *n* = 3 and open uniform knot vector $\vec{t}=\left\{t_{0},t_{1},...,t_{m+n}\right\}$ (0 ⩽ *j* ⩽ *m* + *n*) was used. The interval between two control points was interpolated by dividing it.

#### Subdivision of the Single Vascular Branches

We divided a single branch into multiple branch segments at regular intervals because the segment length could affect quantification, such as branch tortuosity. The remaining segment at the end was discarded if it was too short.

### Model Result Cropping

The two-dimensional numerical model results were cropped into FAZ-centered squares with the side length equal to twice the optic disc–FAZ distance (Supplementary Fig. S4d).

### Image Quantification

We applied the processed OCTA data, as described in the previous section, to the quantifications and branch trajectories of the numerical model results. We used 10 numerical model results under each parameter and ten 12 × 12 mm OCTA data in quantifications.

#### Verticality of the Vessels Around the FAZ

The orientation of each branch segment was measured as the axial angle θ from 0 to $\frac{\pi }{2}$, where the FAZ–optic disc direction was defined as θ = 0. Along the nasal-temporal or superior–inferior axis around FAZ, four regions of interest (ROIs) were defined with the shape of a square with sides of one-half the length of the FAZ-OD distance (Supplementary Fig. S4e, solid yellow boxes: the nasal-temporal horizontal areas, dotted yellow boxes: the superior–inferior vertical areas). The mean angle of branch segments was calculated for each ROI (model: ϕ_model_(*i*, *m*), OCTA: ϕ_OCTA_(*i*, *m*), where *i* and *m* denote the image and ROI number, respectively). As the index of verticality at each ROI, we computed $\sin^{2}\left(\theta_{\text{model}}\left(i,m\right)-\bar{\theta_{\text{OCTA}}}\left(m\right)\right)$, and averaged this index across all ROIs in each simulation.

#### Global Vascular Orientation in Each Area

We used the processed images highlighting vascular structures but not skeletonized for OCTA data (Materials and Methods–OCTA Image Processing) and cropped numerical simulation results for the model (Materials and Methods–Model Result Cropping) in this quantification. First, we divided the images into a 5 × 5 grid (Supplementary Fig. S4f). We applied a two-dimensional fast Fourier transform (FFT) for each region. We calculated the angular profile *I*(θ) by averaging the power spectral amplitude of the frequency components at each angle bin θ. Calculating the weighted average of *I*(θ)^10^ for peak enhancement and adding π/2 to it, we get the vascular direction in each region (model: θ_model_(*i*, *m*), OCTA: θ_*OCTA*_(*i*, *m*), *i*: image number, *m*: territory number). We regarded $\sin^{2}\left(\theta_{\text{model}}\left(i,m\right)-\bar{\theta_{\text{OCTA}}}\left(m\right)\right)$ as the direction discrepancy in each region between the numerical simulations and OCTA data. We also defined the score for each numerical parameter set as its mean across 5 × 5 grid regions.

#### Branch Tortuosity

Before this quantification, we rescaled OCTA images so that their OD–FAZ length matched that in the model. The rescaled OCTA images were processed with the same methods as described above (Materials and Methods–OCTA Image Processing–Vascular Structure Identification, Extraction of Vascular Branch Coordinates, and Subdivision of the Single Vascular Branches). For model results, we also obtained the branch trajectories as described above (Materials and Methods–Model Result Cropping).

For each branch, we defined the tortuosity index as the ratio of the total length to the length between both ends. We then calculated the mean value across all branch segments for each OCTA image or numerical simulation data. Finally, for each numerical simulation parameter set, we computed the parameter score as the difference between the mean score across multiple simulation trials and the mean score across multiple OCTA images.

#### Vascular Density

This quantification was performed using the processed OCTA data (Materials and Methods–OCTA Image Processing–Vascular Structure Identification) and numerical model results (Materials and Methods–Model Result Cropping). We defined the vascular density score as the ratio of the vascularized area to the total area. For a numerical simulation parameter set, we calculated the difference between the mean vascular density obtained from multiple numerical simulations and that from OCTA images.

#### Radiality Around FAZ

In this quantification, we used the processed images highlighting vascular structures but not skeletonized for OCTA data (Materials and Methods–OCTA Image Processing) and cropped numerical simulation results for the model (Materials and Methods–Model Result Cropping). We set four square ROIs of equal size adjacent to the superior, inferior, temporal, and nasal sides of the FAZ region. We applied a two-dimensional FFT to each ROI and measured the angular profile *I*(θ) as previously described. Calculating the weighted average of *I*(θ)^10^ for peak enhancement and adding π/2 to it, we get the vascular direction in each region (model: θ_model_(*i*, *m*), OCTA: θ_*OCTA*_(*i*, *m*), *i*: image number, *m*: territory number) (Supplementary Fig. S4g). We defined the radiality index as cos ^2^(θ_model_(*i*, *m*)) and cos ^2^(θ_OCTA_(*i*, *m*).

#### Radiality Around the Optic Disc

We utilized the processed OCTA data (Materials and Methods–OCTA Image Processing–Subdivision of the Single Vascular Branches) and the numerical model results in this quantification. We detected branch segments near the nasal side of the optic disc. We calculated the angular difference θ_OD_ between the line connecting both ends of each branch segment and the line connecting the optic disc center and the segment midpoint (Supplementary Fig. S4h). We used cos ^2^(θ_OD_) as the radiality index.

#### Characterization of Arcade Vessels

First, we manually tracked the superior–inferior temporal arcades in the processed images, highlighting vascular structures but not skeletonized for OCTA data (Materials and Methods–OCTA Image Processing) and cropped numerical simulation results for the model (Materials and Methods–Model Result Cropping). In each image, we measured three distances between the superior and inferior intersections of the arcade vessels and vertical lines drawn as follows (Supplementary Fig. S4i): 1) a vertical line passing through the center of the FAZ (*l*_FAZ_), 2) a vertical line passing through the midpoint between the optic disc and the FAZ (*l*_nasal_), and 3) a vertical line passing through the point on the temporal side of FAZ, located at a distance equal to the half-length between the FAZ and the optic disc (*l*_temporal_). We calculated three indices: $\frac{l_{\text{nasal}}}{l_{\text{FAZ}}}$, $\frac{l_{\text{temporal}}}{l_{\text{FAZ}}}$, and $\frac{l_{\text{temporal}}}{l_{\text{FAZ}}}-\frac{l_{\text{nasal}}}{l_{\text{FAZ}}}$, which was based on the previous study.^43^

#### Branching Angle

We manually measured branch angles in OCTA images using the angle tool in Fiji.^44^ We measured apparent branch angles in our model results around the FAZ as follows. First, we dilated and skeletonized the output images. We detected the branching points by counting the number of adjacent grid points. We excluded branching points that connected to branches that were too short. From a branching point O, we picked up three points (A, B, and C) located at a fixed distance along each branch, and measured three angles formed by two of these points and the branching position (∠AOB, ∠BOC, ∠COA). The smallest one of these angles was defined as the branching angle.

### Ethics

This work was approved by the Institutional Review Board for Clinical Research at Tokyo Women's Medical University (2023-0095) and Kyushu University (23310-00). This study follows the relevant guidelines and regulations. After an explanation of the study, written informed consent was obtained from all subjects and/or their legal guardians.

## Results

### Comparison of FAZ Formation Mechanisms Using Endothelial Dynamics Models

**Figure 2.**
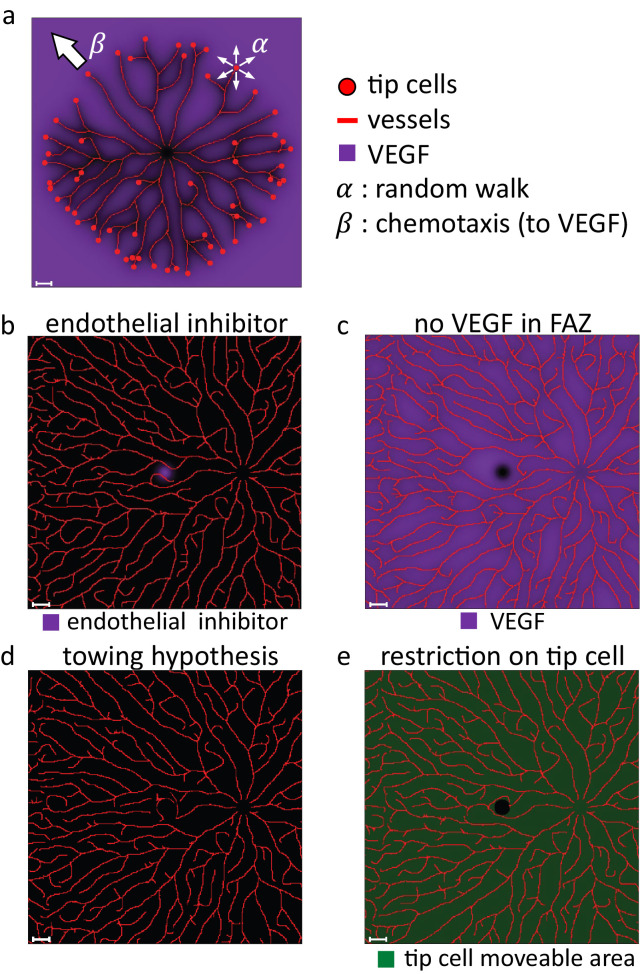
Comparison of hypothetical mechanisms for FAZ formation. (**a**) The schema of forming vascular structures in our model. Tip cells undergo random migration. They also migrate toward VEGF, whose distribution is determined by oxygen supplied from vessels. The coefficients of these effects are α and β, respectively. Vessels form on the paths of the tip cells. (**b–e**) The numerical simulation results are based on candidate mechanisms for FAZ formation. (**b**) Inhibitory molecule hypothesis. (**c**) No chemoattractant hypothesis. (**d**) Towing hypothesis. (**e**) Keep-out hypothesis. *Scale bars*, 1 mm.

To reproduce human retinal vascular development, we assumed that the overall pattern of human retinal vasculature was formed by the angiogenic process, based on observations in previous studies.^15^^,^^16^ In this process, we assumed that tip cells migrate via random walk and chemotaxis, leaving vessel structures along their paths (Fig. 2a; see Mathematical Model–Endothelial Cell Dynamics for the detailed description). Initially, 10 tip cells were evenly positioned around the optic disc. We assumed tip cells migrate toward hypoxic regions, where their chemoattractant, VEGF, is highly expressed.^4^^–^^6^ In our model, oxygen is supplied from preexisting retinal vessels and the central region around the optic disc, which corresponds with the supply from the central retinal artery. The latter prevents tip cells from approaching the center of the optic disc. We note that oxygen is supplied from all endothelial cells, not only tip cells, in our model. Because vascular regression is not implemented in this model, all vessels were assumed to supply oxygen. Similar assumptions about oxygen supply were adopted in several previous studies.^45^^,^^46^ In summary, we modified the simple angiogenic mathematical model with tip cell migrations to align with the human retinal vascular development and incorporated certain retinal geometrical features into our model.

Because we assumed that oxygen dynamics are much faster than migration dynamics, the oxygen distribution was determined at each time step based on the vascular patterns. We also considered that vessels bifurcate by tip cell duplication with constant probability *p*_branch_Δ*t* when the oxygen concentration is below a threshold *u*_branch_.^7^ To prevent excessive angiogenesis in vascularized regions, we also assumed that tip cell motility decreases exponentially with the local VEGF concentration,^34^ which was assumed to be inversely correlated with the local oxygen concentration *u*(*x*, *y*, *t*). We did not consider blood flow-driven vascular remodeling in our model because we aimed to construct the minimal model for human-specific retinal vascular formation, and the formation of large vessels precedes the remodeling process.^15^

Based on the observed vascular pattern in OCTA, we considered several possible hypotheses for the FAZ formation mechanism (see Mathematical Model–FAZ Formation Hypotheses for a detailed description). We first hypothesized that some inhibitory molecules are secreted from the foveal zone (inhibitory molecule hypothesis). We assumed pigment epithelium-derived factor (PEDF) is a candidate for the inhibitory molecule hypothesis. PEDF is secreted by retinal pigmented epithelial cells, especially highly at the developing fovea.^47^ Because PEDF is known as a negative regulator of angiogenesis,^48^ it may prevent endothelial cells from entering the foveal region. However, vessels did not travel toward the foveal zone, but traveled like avoiding the foveal zone (Fig. 2b), which is different from the reality of the human retinal vascular pattern (Fig. 1c).

Next, we assumed that some chemoattractant molecules are absent in the foveal zone (no chemoattractant hypothesis). A different vascular pattern was reproduced under this hypothesis (Fig. 2c). Furthermore, contrary to this hypothesis, the developing fovea in primates expresses a high level of VEGF, a major chemoattractant factor of endothelial cells.^49^

Then, we also considered the possibility that the blood vessel is formed first but later is removed by tissue deformation (towing hypothesis). The towing hypothesis is proposed as a mechanism of foveal pit formation.^50^ In this hypothesis, after a layered construction without a pit is formed at the incipient fovea, ganglion cell bodies are pulled away from the center of the macula by their axons and fall to one side, resulting in the formation of a foveal pit. In this case, vessels were pushed out and did not form a radially inward vascular pattern (Fig. 2d).

Last, we hypothesized that blood vessels are attracted but cannot move into the foveal zone (keep-out hypothesis) (Fig. 2e). Astorcytes, which supported vascular development,^16^^,^^51^^,^^52^ are not present in the developing fovea.^53^ The numerical simulations under this hypothesis partly recapitulated the characteristic vascular pattern toward the center of the fovea in the immediate vicinity of the foveal zone (Fig. 2e). However, the simulated pattern differed from the actual retinal vessels in two ways. First, vessels did not grow toward the foveal zone from the upward and downward sides. Second, we did not observe a vertically facing vascular pattern in numerical simulations, where vessels from the upper and lower sides, toward the horizontal vascular borderline in the temporal region of the FAZ, do not generally invade the other area, forming a vertical interlocking-like structure on the temporal side. Therefore, we focused on keep-out hypothesis and improved this model to reproduce such retinal vessel features.

### Modeling Astrocyte Dynamics

**Figure 3.**
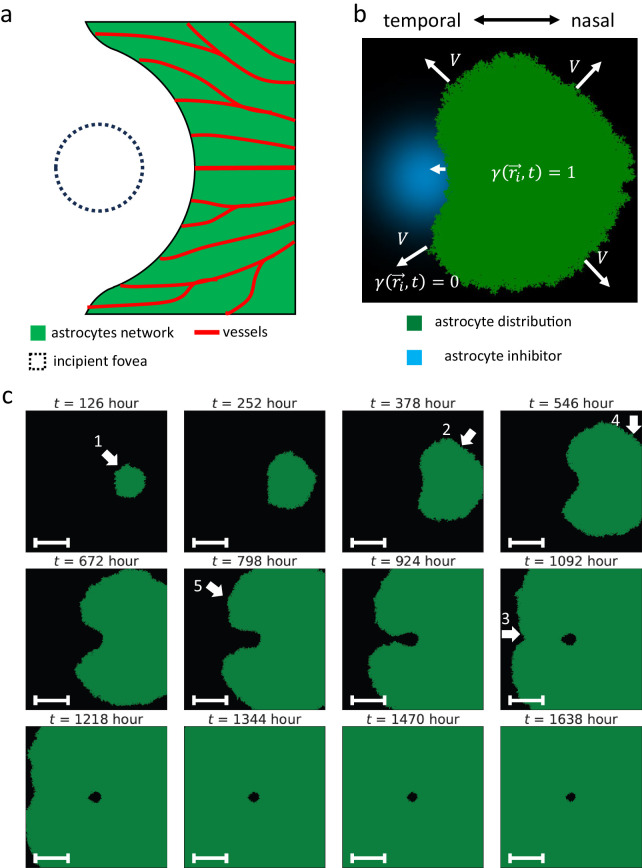
Astrocyte dynamics model. (**a**) The schema of the distribution of astrocytes and vessels during development. (**b**) The schema of the astrocyte dynamics model. (**c**) Time course of the numerical simulation. Arrow 1: Slow-down of astrocyte expansion on the temporal side. Arrow 2: A crescent shape of astrocyte distribution during expansion. Arrow 3: Merge of astrocyte distribution from the superior and inferior sides. Arrow 4: Faster arrival of astrocytes to the nasal periphery than to the temporal one. Arrow 5: A jagged border of the astrocyte distribution. All *scale bars*, 5 mm.

In mouse retinal development, astrocytes grow before angiogenesis.^54^^–^^56^ It appears that astrocytes support vessel elongation.^16^^,^^51^^,^^52^ Some previous reports showed that the front of the growing astrocyte network overlapped with the vascular front and indicated that the astrocytic scaffold spreads concomitant with retinal angiogenesis in some mammals, such as macaque (Fig. 3a,^57^). Moreover, it was also reported that astrocytes support endothelial cells via VEGF, fibronectin, and cell–cell adhesion.^4^^,^^5^^,^^8^^–^^12^ Therefore, in our model, we assumed that the fronts of the astrocyte and endothelial network radially expand concomitantly, and the astrocyte template restricts the angiogenic area.

In addition, several previous studies reported that astrocytes did not distribute at the developing fovea.^53^ We assumed some inhibitory molecules at the fovea inhibit astrocyte expansion. Such inhibitors have not been experimentally identified so far. Still, we speculated that some candidate signaling pathways or molecules, such as the ephrin-Eph system or fibroblast growth factor 8 (FGF8),^58^^,^^59^ which we discuss in detail. We expected the formation of FAZ by assuming the presence of an astrocyte inhibitor.

Because retinal ganglion cell axons guide astrocytes during their expansion,^60^ we modeled the effect of RGCs on retinal astrocyte development. Although we tested a simple mathematical model in which astrocytes migrated along the direction of retinal ganglion cells axons, this model did not reproduce the astrocyte developmental pattern (Supplementary Fig. S5, Supporting Information–Effect of Retinal Ganglion Cell Axons on Astrocyte Migration). This implied that the effect of the retinal ganglion cell axon alone would be insufficient for astrocyte pattern formation.

We assumed that the expansion speed of the astrocyte region decreases with the inhibitor concentration *C*_inhibitor_. Under this assumption, we implemented astrocyte dynamics with a lattice-based model by modifying the Eden growth model^36^ (Fig. 3b, see Mathematical Model–Astrocyte Dynamics for a detailed description). In the Eden growth model, the region expands one square lattice unit on its border at every time step. However, this differs from the actual retinal astrocyte growth, in which the region is considered to grow in all directions simultaneously at a speed of $V=\frac{\Delta x}{\Delta t}P$ (*P*: the expansion probability). In our model, we also considered the case where *P* depends on the inhibitor concentration.

In this model, astrocytes spread out to the space adjacent to the boundary with the probability *P*(*C*_inhibitor_(*x*, *y*)) at every time step. We assumed that the higher the concentration *C*_inhibitor_(*x*, *y*), the lower the probability *P*(*C*_inhibitor_(*x*, *y*)). We can expect that the velocity is reduced around the fovea, and the astrocyte area expands while it avoids and encircles the foveal region.

The astrocyte dynamics model showed that astrocyte distribution avoided the foveal region and gradually spread on the retina (Fig. 3c; Supplementary Video S1). Astrocytes, starting to spread from the optic disc at first, slowed down under the influence of astrocyte migration inhibitor in the temporal side of the optic disc (Fig. 3c, arrow 1). Subsequently, astrocyte distribution avoided the fovea by making a detour around it and formed a crescent shape (Fig. 3c, arrow 2), which was consistent with the observation in the human retina.^57^ After that, they merged from the superior and inferior sides in the temporal zone (Fig. 3c, arrow 3). In contrast, on the nasal side, the influence of the inhibitor was weaker on astrocytes, allowing them to expand radially (Fig. 3c, arrow 2). Because the optic disc was more nasally located than centrally, and foveal side astrocytes decelerated owing to the effect of the inhibitor, astrocytes reached the retinal nasal periphery before the temporal one (Fig. 3c, arrow 4). Additionally, directions of spreading astrocytes corresponded to vessel flow in OCTA imaging (Figs. 1c, 3c). This model also reproduced the jagged boundary of astrocyte distribution in the actual developing retina^57^ (Fig. 3c, arrow 5, and Supplementary Fig. S6). Although this sometimes induced small no-astrocyte “islands” near the astrocyte edge, these spaces were often filled probabilistically at once (Supplementary Video S1). These results indicated that this astrocyte dynamics model reproduced the process of astrocyte area expansion.

### Combined Model of Angiogenesis and Astrocyte Expansion

We considered the relationship between vascular dynamics and astrocyte expansion by combining the FAZ formation model and the astrocyte dynamics model (see Mathematical Model–Endothelial Cell Dynamics for a detailed description). We assumed that vessels can grow only in the region where astrocytes already exist. In Equation (1), when tip cells enter the astrocyte-free zone ($\gamma (\vec{r})=0$), they stop ($\vec{r}\left(i,t+1\right)=\vec{r}\left(i,t\right)$). Therefore, vessels develop only within the astrocyte area, so it was expected that the fronts of astrocytes and vessels would be very close.

This model became a steady state in the region where vascular structures were dense enough (Supplementary Fig. S7; *t* ⩾ 1680), which was induced by the two following factors reacting to high oxygen concentration: a limitation of branching and a deceleration of tip cell migrations.

**Figure 4.**
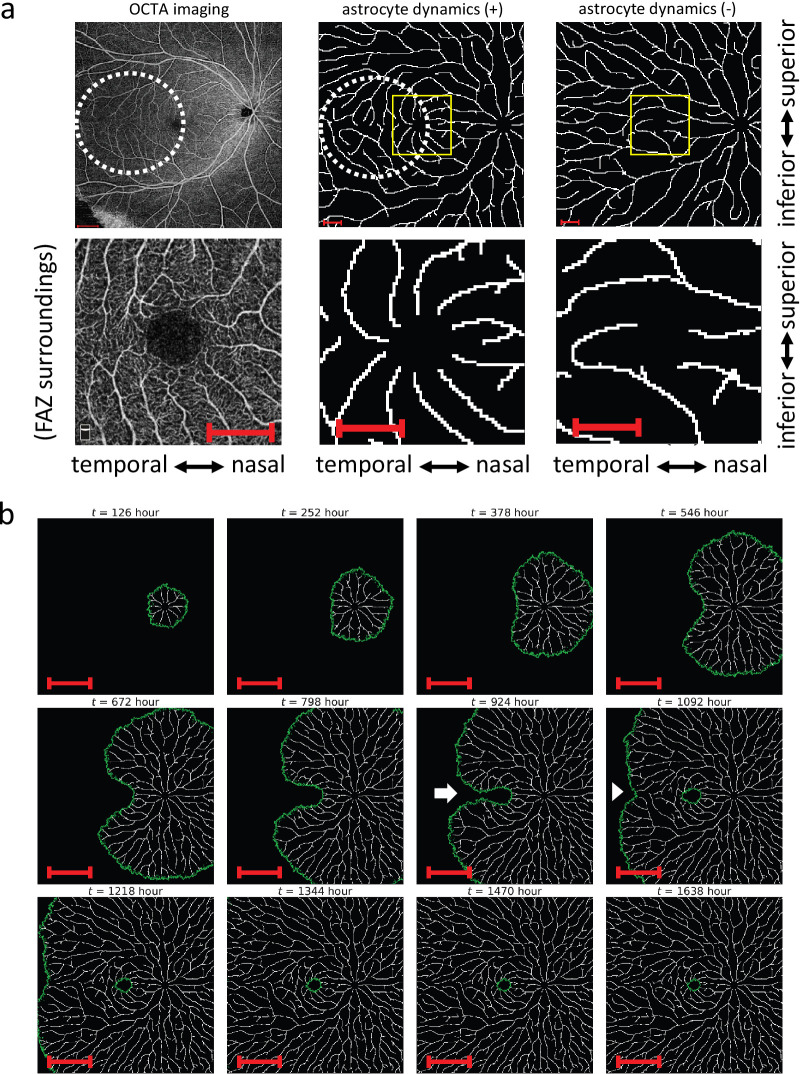
Combined model of angiogenesis and astrocyte expansion. (**a**) The comparison of an OCTA image (*left*) and the numerical simulations with or without astrocyte temporal dynamics (*center*: the combined model, *right*: keep-out model, *top*: overview images, *bottom*: enlarged images corresopnding to yellow squares in the *top*). Note that the enlarged OCTA view is from a different sample than the overview. Dotted white circle: Formation of the vertically facing pattern in the temporal region during development. *Scale bars*, 1 mm. (**b**) Time course of the combined model. *Arrow*: An avascular area that connects the FAZ and the temporal avascular area. *Arrowhead*, ‘notch’ structure similar to that observed in ROP patients. *Scale bars*, 5 mm.

This model reproduced radially inward vascular pattern into the FAZ from all directions (Fig. 4a, lower center), which was not observed in the other models we tested (Figs. 2b–e). Vessels branched from the superior and inferior temporal arcades also faced vertically in that foveal temporal region (Fig. 4a, dotted white circle). In this model, vessels spread radially around the optic disc and far from the fovea (Figs. 4a, 4b; Supplementary Video S2), which also holds in every hypothesis we tested (Figs. 2b–e). In addition, this model reproduced both superior and inferior arcades (Fig. 4a, upper center), which were partially visible in the other hypothesis (Figs. 2b–e). This model reproduced sparser vessels around the optic disc compared to other regions (Fig. 4a; Supplementary Figs. S8a, S8b). This is possibly due to the stochastic formation of new branches, resulting in a local avascular region. Similar events would be prevented in the peripheral areas because other branches can likely fill them among a relatively large number of tip cells compared with the central region. Interestingly, this model also captured the characteristic patterns during development (Supplementary Video S2). This model transiently showed an avascular area that connects FAZ and the temporal avascular area (Fig. 4b, arrow), similar to the actual fetal retina.^61^^,^^62^ Moreover, the model reproduced the recessed border between the vascular and avascular areas at the temporal side of FAZ during development. This pattern is similar to the structure known as the notch in some retinopathy of prematurity (ROP) patients (Fig. 4b, arrowhead).^63^ Because normal retinal vascular development was arrested in ROP, a notch-like structure may also be present in the normal fetal retina. Taken together, the vascular pattern in this model was qualitatively similar to the human retinal pattern. We also compared our model (astrocyte dynamics (+)) with that without astrocyte dynamics (astrocyte dynamics (−)), in which astrocyte distribution was temporally fixed as the final pattern in the combined model. This model without astrocyte dynamics did not reproduce the characteristic vascular patterns around FAZ (Fig. 4a, right).

**Figure 5.**
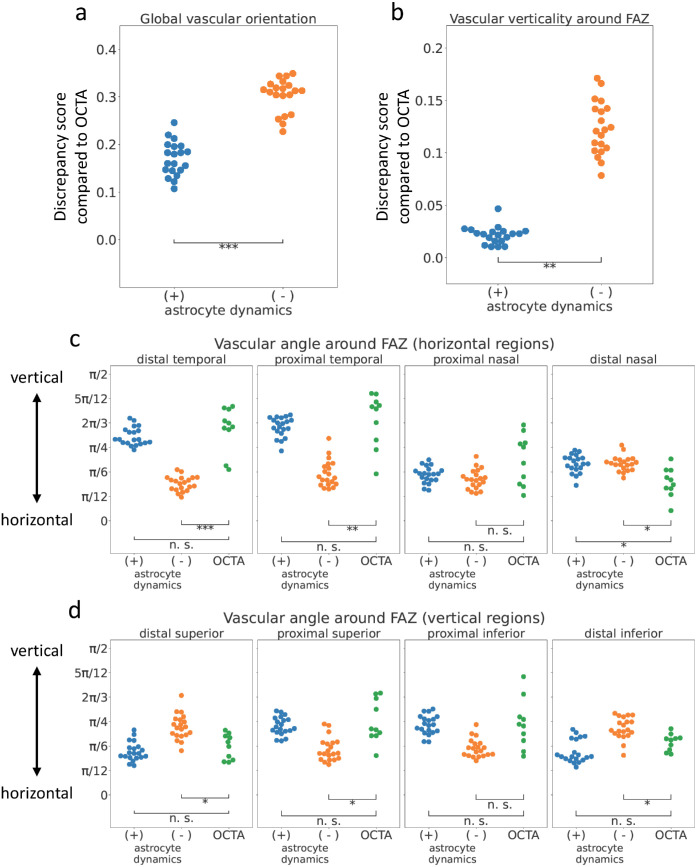
Quantitative comparison of vascular patterns among the models with and without astrocyte dynamics and the OCTA data. (**a**) Total score of the global vascular orientation compared to OCTA data. (**b**) The total score of the vascular verticality around the FAZ compared with OCTA data. (**c, d**) The vascular verticality around the FAZ in each region. (**c**) Four temporal-nasal horizontal regions. (**d**) Four superior-inferior vertical regions. * *P* < 0.01; ** *P* < 0.001; *** *P* < 0.0001 (Welch's *t* test, mathematical models: *N* = 20, OCTA data: *N* = 10).

We quantitatively compared the features of the vascular pattern in our model results and OCTA images. Global vascular orientation, calculated using two-dimensional FFT, of the astrocyte dynamics (+) model was closer to OCTA images than that of the astrocyte dynamics (−) model (Fig. 5a; Supplementary Fig. S9, Materials and Methods—Image quantification—Global Vascular Orientation in Each Area). We also quantitatively assessed vessel orientation in several ROIs around the FAZ in both numerical simulations and OCTA images (Figs. 5b–d). In general, the astrocyte dynamics (+) model reproduced a similar vascular pattern to OCTA data in this aspect (Fig. 5b). In more detail, horizontal vessel orientation was observed on the nasal side in the astrocyte dynamics (+) model and OCTA images, reflecting vessels running from the optic disc to the fovea (Fig. 5c). On the temporal side of the FAZ, vessels were more vertical, and this tendency was stronger, especially near the FAZ, indicating that this model quantitatively reproduced characteristic vessel orientation patterns around the FAZ. On both the superior and inferior sides of the FAZ, the closer part to the FAZ showed a more vertically aligned pattern (Fig. 5d). However, the astrocyte dynamics (−) model did not reproduce these features, indicating that the vascular pattern around FAZ of the astrocyte dynamics (+) model is more similar to OCTA images (Figs. 5b–d). In all astrocyte dynamics (+) models and the OCTA images, the radiality of the vascular pattern around FAZ was higher in the regions except the temporal side, where it was lower due to the vertically oriented vascular pattern (Supplementary Fig. S10a). The vascular structures around the optic disc exhibited a radial pattern in both models and in the OCTA images (Supplementary Fig. S10b). Both models with and without the astrocyte dynamics reproduced the vascular patterns around the optic disc that were slightly different from the OCTA data (Fig. 5c distal nasal, Supplementary Fig. S10b). This discrepancy may be attributed to the simplification of the vascular structures around the optic disc in our model. The arcade vessels in the astrocyte dynamics (+) model more closely resembled that those in the OCTA images than those in the astrocyte dynamics (−) model do, indicating similarily curved strcuture in the astrocyte dynamics (+) model and the OCTA images (Supplementary Figs. S10c, S10d). In summary, the presence or absence of astrocyte dynamics has a significant impact on vascular patterns around the fovea.

## Discussion

In this study, we first considered four hypotheses (Figs. 2b–e). Because all of them failed to reproduce the actual vascular patterns, we then focused on the retinal astrocyte function as an angiogenic scaffold. When we assumed the astrocytes spread along with the development of the vascular network and restricted the tip cell migration area, the model resulted in similar vascular patterns to OCTA images, especially around the fovea (Figs. 4a, 5). Our model reproduced a radially outward vascular pattern from the optic disc, inferior and superior temporal arcades, a radially inward vascular pattern around FAZ, and a vertically oriented pattern toward the horizontal vascular boundary in the temporal region of FAZ. Moreover, the linear avascular area and the notch-like structure in the middle of the development in the model (Fig. 4b, arrow, arrowhead), which was observed in several previous studies.^61^^–^^63^ These results suggest that astrocyte dynamics, coupled with vascular development, are crucial for establishing the proper vascular patterns unique to human retinas.

However, even if we do not assume the time dependence of astrocytes, tip cells travel radially in the retinal peripheral region. This finding suggests that astrocyte dynamics, concomitant with vascular development, predominantly control vascular patterns around the fovea, but not the radial patterns in the periphery (Fig. 4a). In fact, the vascular pattern in our model is similar to the astrocyte expansion direction, especially around FAZ (Supplementary Fig. S14, Supporting Information–Calculation of the Vector Field of the Astrocyte Expansion). Furthermore, this vascular pattern formation did not occur either in the absence of chemotaxis (β = 0 mm^5^/hour; Supplementary Fig. S15a) or under weak chemotactic effects (β = 2.682 × 10^−6^ mm^5^/hour; Supplementary Fig. S15b). These results indicate that tip cells must catch up with the front of the astrocyte distribution to reproduce the actual vascular pattern through negative chemotaxis away from oxygen gradients. Retinal astrocytes are known to provide a scaffold for tip cell migration^9^^–^^11^ and expand together with the vascular network in the human retina.^57^ Interestingly, these two factors caused retinal astrocyte dynamics to have chemotaxis-like effects on tip cell movement, especially around the FAZ.

Our mathematical model also provided the possible time course of human retinal vascular development (Fig. 4b; Supplementary Video S2), which is difficult to observe directly in vivo. The simulated time course corresponded to the previous observations in several developmental stages.^16^ Additionally, the model can be applied to investigate abnormal situations at a low cost. For example, our model has the potential to simulate the process and mechanism of retinal vascular developmental disorders, such as foveal hypoplasia, by adjusting certain parameters. The numerical simulation with very large *u*_branch_, at which branching could happen under relatively hyperoxic conditions, reproduced a very dense vascular pattern (Supplementary Fig. S16), which resembled neovascularization observed near the ridge in ROP.^63^ Moreover, our model is expandable to incorporate astrocyte death induced by hyperoxia.^64^ Therefore, mathematical modeling is beneficial for studying human retinal development, and our model could serve as a basis for further elucidation.

In our model, vessels around the optic disc sometimes were sparser than ones in other regions (Figs. 4a, 4b; Supplementary Fig. S8), which is considered to be caused by a lower probability that some branches fill avascular regions because of fewer tip cells around the optic disc. We speculated that this relates to the functions of radial peripapillary capillaries. Radial peripapillary capillaries are located in the superficial retinal nerve fiber layer, around the optic disc in the human retina.^22^^,^^65^^,^^66^ One of their roles might be supplying oxygen and nutrition by complementing the sparse region around the optic disc.

There are several candidate molecules of astrocyte inhibitors that inhibited astrocyte progression around the fovea in our model (see Results-Modeling Astrocyte Dynamics). One candidate is Eph-A6, a membrane receptor localized in the ganglion cell layer around the fovea.^58^ In addition, Pax2-positive astrocytes, which are considered immature and motile, express ephrin-1 and -4, which are known as Eph ligands. Because ephrin-Eph induces bidirectional signaling and repulsive effects,^67^^–^^69^ it is possible that Eph plays a role as an astrocyte inhibitor by repelling ephrin-positive astrocytes. The other candidate is FGF8. It is reported that Fgf8 is expressed around a central high-acuity area in the chick retina, which corresponds with the fovea.^59^ They suggested that retinoic acid negatively regulates Fgf8 expression to form a central high-acuity area. FGF8 may inhibit astrocyte migration in the human retina.

Our model may apply to other aspects of retinal patterns in the future. For example, pathological situations, such as ROP, diabetic retinopathy, and familial exudative vitreoretinopathy, can be incorporated into the model. Our model showed several similarities to the vascular pattern observed in ROP, including a notch-like structure (Fig. 4b, arrowhead) and a very dense vascular pattern (Supplementary Fig. S16). Moreover, this model may be applicable to future studies on disease mechanisms, such as diabetic retinopathy and familial exudative vitreoretinopathy, which involve vascular degeneration and expansion of the angiogenic range. In this study, we did not consider oxygen supply from sources other than retinal vessels and retinal vascular multilayering. Although these are important aspects of retinal vascular pattern formation, we focused on the pattern formation of larger vessels formed by angiogenesis. We also did not consider the flow-induced vascular remodeling process and vessel thickness. We may expand our model by integrating these factors to reproduce the remodeling process and the resultant hierarchical vascular structure. We compared the simulation results of fetal retinal vascular development with adult OCTA images, rather than actual fetal vascular patterns, because they cannot be directly observed. Although there is little difference between adult and perinatal patterns of larger retinal vessels,^16^ it remains unclear whether the smaller vessel patterns in actual fetal retinas differ from those in adult OCTA images and our model. It may be interesting to combine some of these components with our model to more realistically reproduce the vascular patterns.

## Conclusions

We developed a mathematical model reproducing human retinal vascular patterns and their developmental processes. The essential assumption was retinal astrocyte dynamics coupled with tip cell movement. This implies that astrocytes contribute to human-specific vascular pattern formation. Our model will provide valuable insights for further investigation into retinal vascular development and disorders.

## Supplementary Material

Supplement 1

Supplement 2

Supplement 3

Supplement 4

Supplement 5

## References

[1] Gariano RF, Gardner TW. Retinal angiogenesis in development and disease. *Nature*. 2004; 438: 960–966. 10.1038/nature04482 16355161

[2] Fruttiger M. Development of the retinal vasculature. *Angiogenesis*. 2007; 10: 77–88. 17322966 10.1007/s10456-007-9065-1

[3] Watanabe T, Raff MC. Retinal astrocytes are immigrants from the optic nerve. *Nature*. 1988; 332: 834–837. 3282180 10.1038/332834a0

[4] Stone J, Itin A, Alon T, et al. Development of retinal vasculature is mediated by hypoxia-induced vascular endothelial growth factor (VEGF) expression by neuroglia. *J Neurosci*. 1995; 15: 4738–4747. 7623107 10.1523/JNEUROSCI.15-07-04738.1995 PMC6577882

[5] Pierce EA. Regulation of vascular endothelial growth factor by oxygen in a model of retinopathy of prematurity. *Arch Ophthalmol*. 1996; 114: 1219. 8859081 10.1001/archopht.1996.01100140419009

[6] Provis JM, Leech J, Diaz CM, Penfold Pl, Stone J, Keshet E. Development of the human retinal vasculature: cellular relations and VEGF expression. *Exp Eye Res*. 1997; 65: 555–568. 9464188 10.1006/exer.1997.0365

[7] Gerhardt H, Golding M, Fruttiger M, et al. VEGF guides angiogenic sprouting utilizing endothelial tip cell filopodia. *J Cell Biol*. 2003; 161: 1163–1177. 12810700 10.1083/jcb.200302047 PMC2172999

[8] West H, Richardson WD, Fruttiger M. Stabilization of the retinal vascular network by reciprocal feedback between blood vessels and astrocytes. *Development*. 2005; 132: 1855–1862. 15790963 10.1242/dev.01732

[9] Jiang B, Liou GI, Behzadian MA, Caldwell RB. Astrocytes modulate retinal vasculogenesis: effects on fibronectin expression. *J Cell Sci*. 1994; 107: 2499–2508. 7844167 10.1242/jcs.107.9.2499

[10] Uemura A. Tlx acts as a proangiogenic switch by regulating extracellular assembly of fibronectin matrices in retinal astrocytes. *J Clin Invest*. 2006; 116: 369–377. 16424942 10.1172/JCI25964 PMC1332029

[11] Stenzel D, Lundkvist A, Sauvaget D, et al. Integrin-dependent and -independent functions of astrocytic fibronectin in retinal angiogenesis. *Development*. 2011; 138: 4451–4463. 21880786 10.1242/dev.071381 PMC3177315

[12] Dorrell MI, Aguilar E, Friedlander M. Retinal vascular development is mediated by endothelial filopodia, a preexisting astrocytic template and specific R-cadherin adhesion. *Invest Ophthalmol Vis Sci*. 2002; 43: 3500–3510. 12407162

[13] Duke-Elder SS . *System of Ophthalmology, Vol. I. The eye in evolution*. Henry Kimpton; 1958.

[14] Duke-Elder SS . *System of ophthalmology, Vol. III, Normal and abnormal development, part 2, congenital deformities*. Henry Kimpton; 1963.

[15] Chan-Ling T. In: *Development of the retinal vasculature*. New York: Academic Press; 2010. p. 22–33.

[16] Provis J. Development of the primate retinal vasculature. *Prog Retinal Eye Res*. 2001; 20: 799–821. 10.1016/s1350-9462(01)00012-x 11587918

[17] Duke-Elder SS, Wybar KC. *System of ophthalmology, Vol. II. The anatomy of the visual system*. Henry Kimpton; 1961.

[18] Adams DL, Precise Horton JC. A Retinotopic map of primate striate cortex generated from the representation of angioscotomas. *J Neurosci*. 2003; 23: 3771–3789. 12736348 10.1523/JNEUROSCI.23-09-03771.2003 PMC6742198

[19] Bringmann A, Syrbe S, Görner K, et al. The primate fovea: structure, function and development. *Prog Retinal Eye Res*. 2018; 66: 49–84. 10.1016/j.preteyeres.2018.03.006 29609042

[20] Gariano RF. Special features of human retinal angiogenesis. *Eye*. 2010; 24: 401–407. 20075971 10.1038/eye.2009.324

[21] Distler C, Weigel H, Hoffmann KP. Glia cells of the monkey retina. I. Astrocytes. *J Comp Neurol*. 1993; 333: 134–147. 8340493 10.1002/cne.903330111

[22] Spaide RF, Fujimoto JG, Waheed NK, Sadda SR, Staurenghi G. Optical coherence tomography angiography. *Prog Retinal Eye Res*. 2018; 64: 1–55. 10.1016/j.preteyeres.2017.11.003 PMC6404988 29229445

[23] Anderson AR, Chaplain MAJ. Continuous and discrete mathematical models of tumor-induced angiogenesis. *Bull Math Biology*. 1998; 60: 857–899. 10.1006/bulm.1998.0042 9739618

[24] Stokes CL, Lauffenburger DA. Analysis of the roles of microvessel endothelial cell random motility and chemotaxis in angiogenesis. *J Theoret Biol*. 1991; 152: 377–403. 1721100 10.1016/s0022-5193(05)80201-2

[25] Pettet GJ, Byrne HM, Mcelwain DLS, Norbury J. A model of wound-healing angiogenesis in soft tissue. *Math Biosci*. 1996; 136: 35–63. 8755336 10.1016/0025-5564(96)00044-2

[26] Tong S, Yuan F. Numerical simulations of angiogenesis in the cornea. *Microvasc Res*. 2001; 61: 14–27. 11162192 10.1006/mvre.2000.2282

[27] Plank MJ. A reinforced random walk model of tumour angiogenesis and anti-angiogenic strategies. *Math Med Biol*. 2003; 20: 135–181. 14636027 10.1093/imammb/20.2.135

[28] Stéphanou A, McDougall SR, Anderson ARA, Chaplain MAJ. Mathematical modelling of flow in 2D and 3D vascular networks: applications to anti-angiogenic and chemotherapeutic drug strategies. *Math Computer Model*. 2005; 41: 1137–1156.

[29] McDougall SR, Anderson ARA, Chaplain MAJ. Mathematical modelling of dynamic adaptive tumour-induced angiogenesis: clinical implications and therapeutic targeting strategies. *J Theoret Biol*. 2006; 241: 564–589. 16487543 10.1016/j.jtbi.2005.12.022

[30] McDougall SR, Watson MG, Devlin AH, Mitchell CA, Chaplain MAJ. A hybrid discrete-continuum mathematical model of pattern prediction in the developing retinal vasculature. *Bull Math Biol*. 2012; 74: 2272–2314. 22829182 10.1007/s11538-012-9754-9

[31] Aghamirmohammadali SMA, Boozarjomehry RB, Abdekhodaie M. Modelling of retinal vasculature based on genetically tuned parametric L-system. *Roy Soc Open Sci*. 2018; 5. 10.1098/rsos.171639 PMC5990753 29892362

[32] Mada J, Tokihiro T. Pattern formation of vascular network in a mathematical model of angiogenesis. *Japan J Indust Appl Math*. 2022; 39: 351–384.

[33] Watson MG, McDougall SR, Chaplain MAJ, Devlin AH, Mitchell CA. Dynamics of angiogenesis during murine retinal development: a coupled in vivo and in silico study. *J Roy Soc Interface*. 2012; 9: 2351–2364. 22438490 10.1098/rsif.2012.0067 PMC3405746

[34] Rousseau S, Houle F, Landry J, Huot J. p38 MAP kinase activation by vascular endothelial growth factor mediates actin reorganization and cell migration in human endothelial cells. *Oncogene*. 1997; 15: 2169–2177. 9393975 10.1038/sj.onc.1201380

[35] Stevens A, Othmer HG. Aggregation, blowup, and collapse: the ABC's of taxis in reinforced random walks. *SIAM Journal on Applied Mathematics*. 1997; 57(4): 1044–1081.

[36] Eden M. A Two-dimensional growth process. *Dynamics of fractal surfaces*. 1961; 4: 223–239.

[37] Distler C, Kopatz K, Telkes I. Developmental changes in astrocyte density in the macaque perifoveal region. *Eur J Neurosci*. 2000; 12: 1331–1341. 10762362 10.1046/j.1460-9568.2000.00029.x

[38] van Rossum G, Drake FL. *Python 3 Reference Manual*. CreateSpace; 2009.

[39] Harris CR, Millman KJ, van der Walt SJ, et al. Array programming with NumPy. *Nature*. 2020; 585(7825): 357–362. 32939066 10.1038/s41586-020-2649-2 PMC7759461

[40] Virtanen P, Gommers R, Oliphant TE, et al. SciPy 1.0: fundamental algorithms for scientific computing in Python. *Nat Meth*. 2020; 17: 261–272. 10.1038/s41592-019-0686-2 PMC7056644 32015543

[41] Bradski G. The OpenCV Library. *Dr Dobb's Journal of Software Tools*. 2000; 25(11): 120–125.

[42] Meijering E, Jacob M, Sarria JCF, Steiner P, Hirling H, Unser M. Design and validation of a tool for neurite tracing and analysis in fluorescence microscopy images. *Cytometry Part A*. 2004; 58A: 167–176. 10.1002/cyto.a.20022 15057970

[43] Nowroozzadeh MH, Moshksar S, Azimi A, Rasti A, Sedaghat A. Comparison of retinal vascular arcade trajectory between eyes with an idiopathic macular hole and the healthy fellow eye. *Int Ophthalm*. 2022; 42: 2219–2225. 10.1007/s10792-022-02221-9 35041130

[44] Schindelin J, Arganda-Carreras I, Frise E, et al. Fiji: an open-source platform for biological-image analysis. *Nat Meth*. 2012; 9: 676–682. 10.1038/nmeth.2019 PMC3855844 22743772

[45] Secomb TW, Hsu R, Park EYH, Dewhirst MW. Green's function methods for analysis of oxygen delivery to tissue by microvascular networks. *Ann Biomed Engineer*. 2004; 32: 1519–1529. 10.1114/b:abme.0000049036.08817.44 15636112

[46] Flandoli F, Leocata M, Ricci C. The Mathematical modeling of cancer growth and angiogenesis by an individual based interacting system. *J Theoret Biol*. 2023; 562: 111432. 36746298 10.1016/j.jtbi.2023.111432

[47] Kozulin P, Natoli R, O'Brien KMB, Madigan MC, Provis JM. The cellular expression of antiangiogenic factors in fetal primate macula. *Invest Ophthalmol Vis Sci*. 2010; 51: 4298–4306. 20357200 10.1167/iovs.09-4905

[48] Dawson DW, Volpert OV, Gillis P, et al. Pigment epithelium-derived factor: a potent inhibitor of angiogenesis. *Science*. 1999; 285: 245–248. 10398599 10.1126/science.285.5425.245

[49] Sandercoe TM, Geller SF, Hendrickson AE, Stone J, Provis JM. VEGF expression by ganglion cells in central retina before formation of the foveal depression in monkey retina: evidence of developmental hypoxia. *J Comp Neurol*. 2003; 462: 42–54. 12761823 10.1002/cne.10705

[50] Essen DCV. A tension-based theory of morphogenesis and compact wiring in the central nervous system. *Nature*. 1997; 385: 313–318. 9002514 10.1038/385313a0

[51] Gariano RF, Sage EH, Kaplan HJ, Hendrickson AE. Development of astrocytes and their relation to blood vessels in fetal monkey retina. *Invest Ophthalmol Vis Sci*. 1996; 37: 2367–2375. 8933753

[52] Selvam S, Kumar T, Fruttiger M. Retinal vasculature development in health and disease. *Prog Retinal Eye Res*. 2018; 63: 1–19. 10.1016/j.preteyeres.2017.11.001 29129724

[53] Provis JM, Sandercoe T, Hendrickson AE. Astrocytes and blood vessels define the foveal rim during primate retinal development. *Invest Ophthalm Vis Sci*. 2000; 41. 10967034

[54] Fruttiger M. Development of the mouse retinal vasculature: angiogenesis versus vasculogenesis. *Invest Ophthalmol Vis Sci*. 2002; 43: 522–527. 11818400

[55] Stone J, Dreher Z. Relationship between astrocytes, ganglion cells and vasculature of the retina. *J Comp Neurol*. 1987; 255: 35–49. 3819008 10.1002/cne.902550104

[56] Ling T, Mitrofanis J, Stone J. Origin of retinal astrocytes in the rat: evidence of migration from the optic nerve. *J Comp Neurol*. 1989; 286: 345–352. 2768562 10.1002/cne.902860305

[57] Provis J. Ontogeny of the primate fovea: a central issue in retinal development. *Prog in Neurobiol*. 1998; 54: 549–581. 10.1016/s0301-0082(97)00079-8 9550191

[58] Kozulin P, Natoli R, Madigan MC, O'Brien KMB, Provis JM. Gradients of Eph-A6 expression in primate retina suggest roles in both vascular and axon guidance. *Mol Vis*. 2009; 15: 2649–2662. 20011078 PMC2791039

[59] da Silva S, Cepko CL. Fgf8 expression and degradation of retinoic acid are required for patterning a high-acuity area in the retina. *Dev Cell*. 2017; 42: 68–81.e6. 28648799 10.1016/j.devcel.2017.05.024 PMC5798461

[60] O'Sullivan ML, Puñal VM, Kerstein PC, et al. Astrocytes follow ganglion cell axons to establish an angiogenic template during retinal development. *Glia*. 2017; 65: 1697–716. 28722174 10.1002/glia.23189 PMC5561467

[61] Chen X, Viehland C, Tran-Viet D, et al. Capturing macular vascular development in an infant with retinopathy of prematurity. *JAMA Ophthalmol*. 2019; 137:1083. 10.1001/jamaophthalmol.2019.2165 PMC6933090 31246250

[62] Provis JM, Hendrickson AE. The foveal avascular region of developing human retina. *Arch Ophthalmol*. 2008; 126: 507. 18413520 10.1001/archopht.126.4.507

[63] Chiang MF, Quinn GE, Fielder AR, et al. International classification of retinopathy of prematurity, third edition. *Ophthalmology*. 2021; 128: e51–e68. 34247850 10.1016/j.ophtha.2021.05.031 PMC10979521

[64] Bucher F, Stahl A, Agostini HT, Martin G. Hyperoxia causes reduced density of retinal astrocytes in the central avascular zone in the mouse model of oxygen-induced retinopathy. *Mol Cell Neurosci*. 2013; 56: 225–233. 23756201 10.1016/j.mcn.2013.06.001

[65] Henkind P. Radial peripapillary capillaries of the retina. I. Anatomy: human and comparative. *Br J Ophthalmolo*. 1967; 51: 115–123. 10.1136/bjo.51.2.115 PMC506341 4959937

[66] Mase T, Ishibazawa A, Nagaoka T, Yokota H, Yoshida A. Radial peripapillary capillary network visualized using wide-field montage optical coherence tomography angiography. *Invest Opthalmol Vis Sci*. 2016; 57: OCT504. 10.1167/iovs.15-18877 27454659

[67] Drescher U, Kremoser C, Handwerker C, Löschinger J, Noda M, Bonhoeffer F. In vitro guidance of retinal ganglion cell axons by RAGS, a 25 kDa tectal protein related to ligands for Eph receptor tyrosine kinases. *Cell*. 1995; 82: 359–370. 7634326 10.1016/0092-8674(95)90425-5

[68] Cheng HJ, Nakamoto M, Bergemann AD, Flanagan JG. Complementary gradients in expression and binding of ELF-1 and Mek4 in development of the topographic retinotectal projection map. *Cell*. 1995; 82: 371–381. 7634327 10.1016/0092-8674(95)90426-3

[69] Nakamoto M, Cheng HJ, Friedman GC, et al. Topographically specific effects of ELF-1 on retinal axon guidance in vitro and retinal axon mapping in vivo. *Cell*. 1996; 86: 755–766. 8797822 10.1016/s0092-8674(00)80150-6

[70] Iizuka O, Kawamura S, Tero A, Uemura A, Miura T. Remodeling mechanisms determine size distributions in developing retinal vasculature. *PLoS One*. 2020; 15: e0235373. 33052908 10.1371/journal.pone.0235373 PMC7556457

[71] Colello RJ, Guillery RW. The early development of retinal ganglion cells with uncrossed axons in the mouse: retinal position and axonal course. *Development*. 1990; 108: 515–523. 2340812 10.1242/dev.108.3.515

[72] Vrabec F. The temporal raphe of the human retina. *Am J Ophthalmol*. 1966; 62: 926–938. 4162879 10.1016/0002-9394(66)91920-9

[73] Yamauchi K, Mizushima S, Tamada A, Yamamoto N, Takashima S, Murakami F. FGF8 signaling regulates growth of midbrain dopaminergic axons by inducing semaphorin 3F. *J Neurosci*. 2009; 29: 4044–4055. 19339600 10.1523/JNEUROSCI.4794-08.2009 PMC6665371

[74] Kolpak A, Zhang J, Bao ZZ. Sonic Hedgehog has a dual effect on the growth of retinal ganglion axons depending on its concentration. *J Neurosci*. 2005; 25: 3432–3441. 15800198 10.1523/JNEUROSCI.4938-04.2005 PMC1564194

