# Supplementary material for: Mathematical Modeling of Human Retinal Vascular Pattern Around the Foveal Avascular Zone
Source: Transl Vis Sci Technol. 2026 Mar 2;15(3):1. doi: 10.1167/tvst.15.3.1 (PMC12967130; doi:10.1167/tvst.15.3.1)
Supplement: Supplement 1 [file tvst-15-3-1_s001.pdf]

102 **Supporting figures**

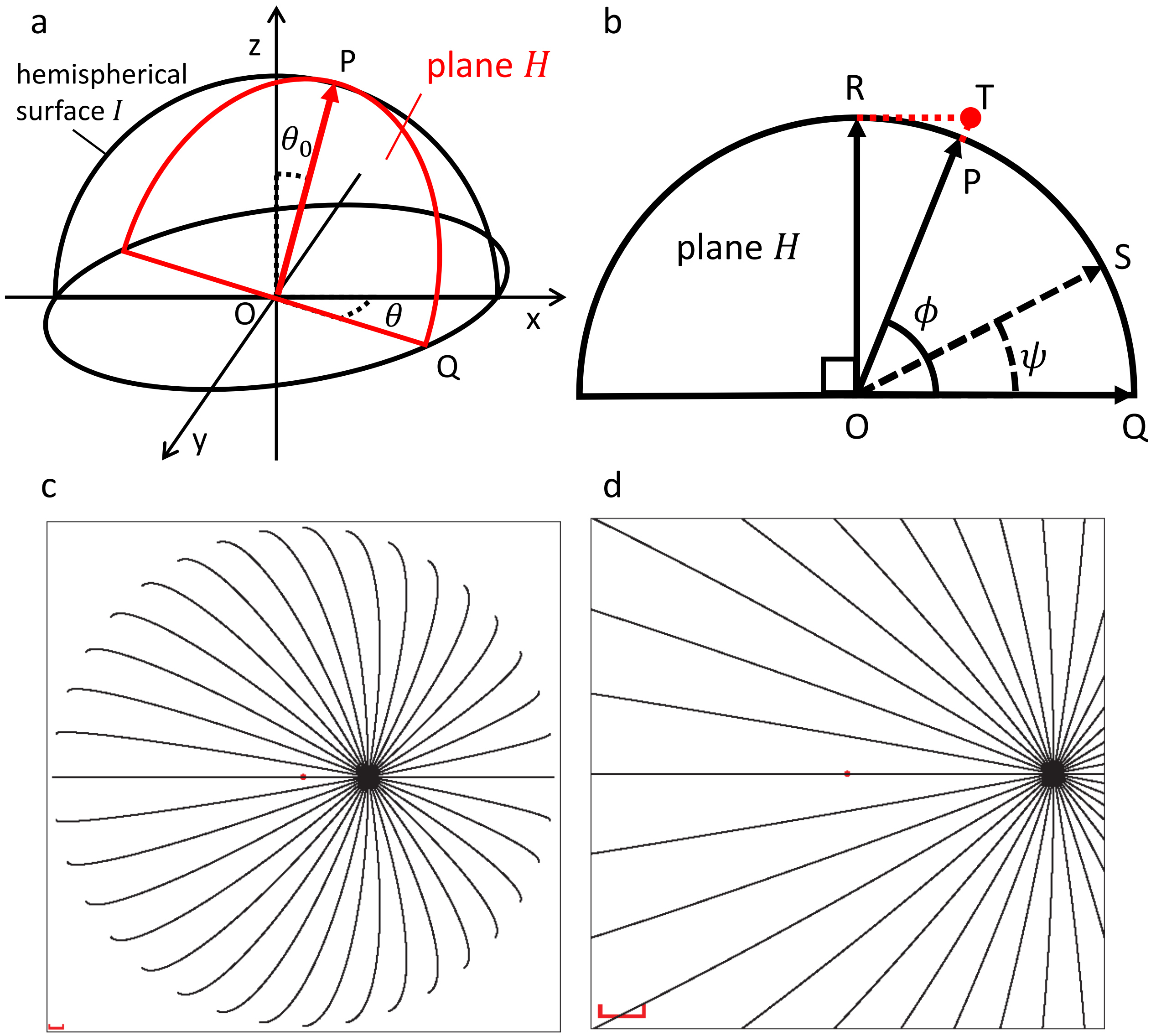

Figure S1. The influence of distortion due to the projection of the retinal spherical surface onto flat vascular images. (a-b) Schemas for the calculation of projection. O: the center of the eye, P: OD position, Q: the edge point of the retina along the trajectory, R: the highest point along the trajectory, S: the evaluation point for calculating the trajectory, T: the point on the extension of line OP which lies at the vertical level as R, I: the hemisphere surface corresponding to the retina, H: the plane containing the trajectory we consider. (c-d) The estimation of projection of radially elongated vessels from the optic disc. (d) was magnified for comparison with OCTA images. Red points: the center points corresponding to the center of the fovea. Scale bars: 1 mm.

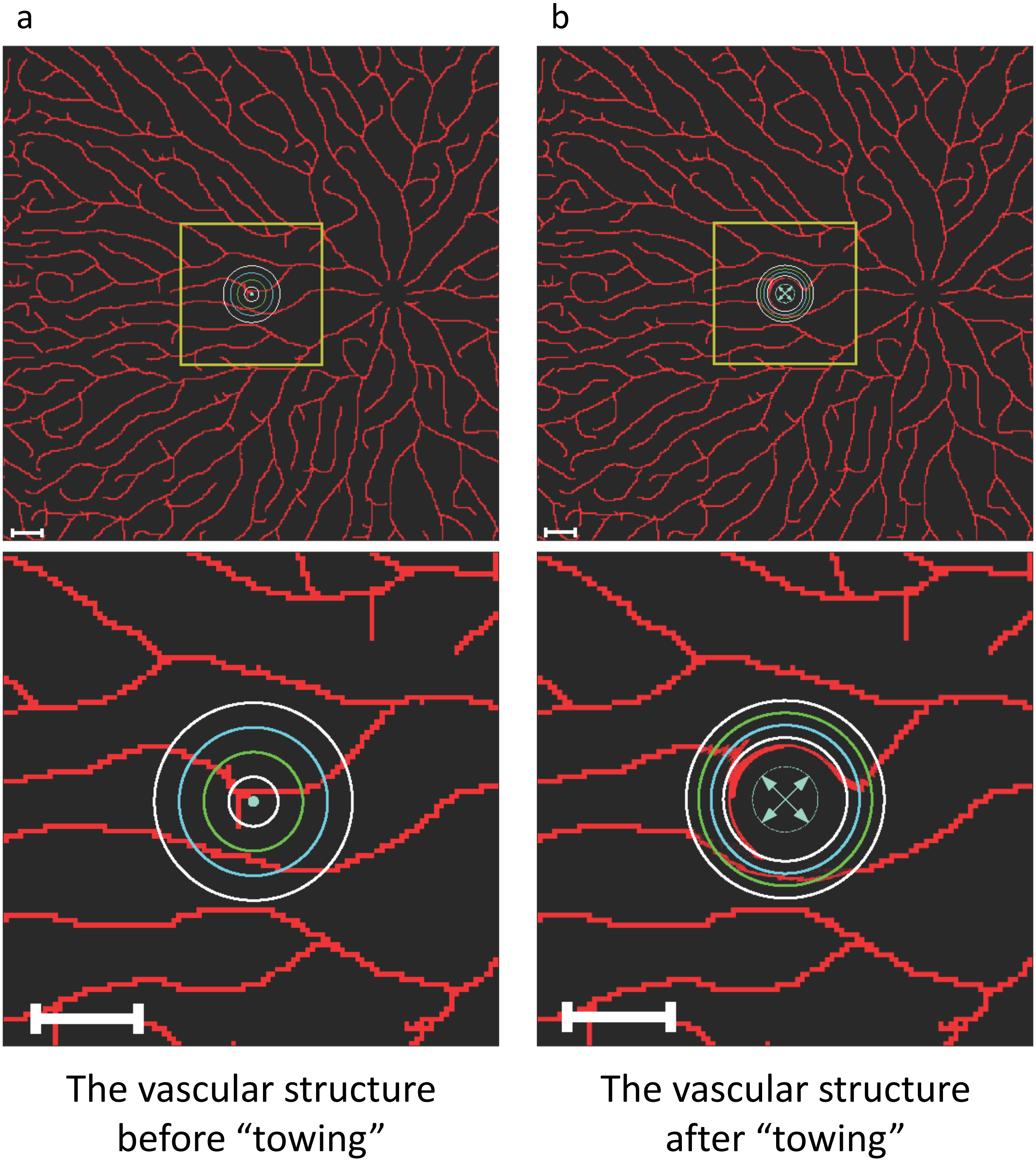

Figure S2. The schematic representation of the towing hypothesis-based model. Numerical simulation results (a) before ”towing,” and (b) after ”towing.” Concentric circles: the correspondence of deformations around FAZ. Top: overview images, bottom: enlarged images corresponding to the yellow squares in each top panel. Scale bars: 1 mm.

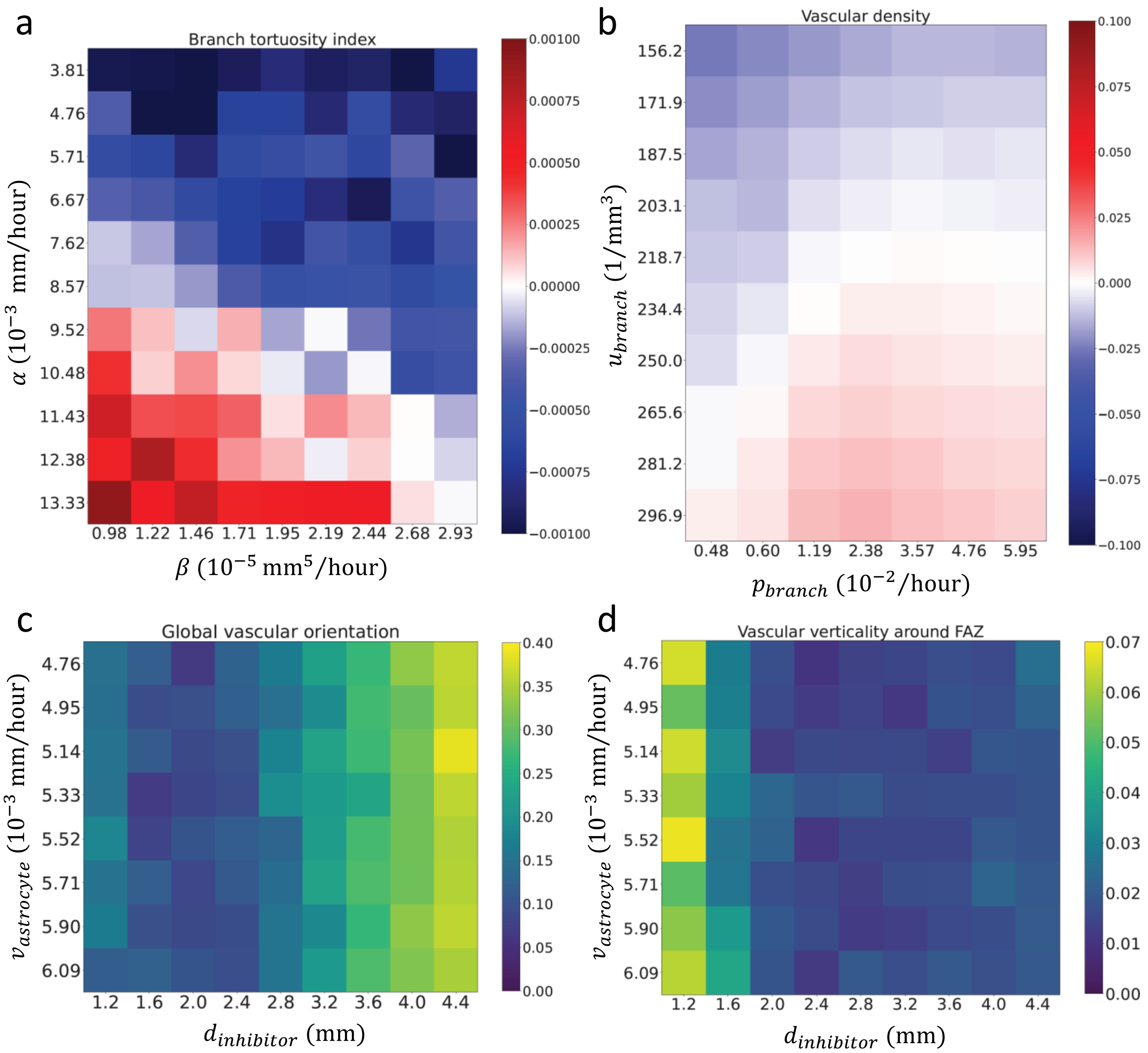

Figure S3. Morphological quantification for the parameter selection. (a) The heat map of the vessel tortuosity index for various α and β, calculated as the difference between the mean score across multiple simulation trials and the mean score across multiple OCTA images. (b) The heat map of the vascular density for various u_{branch} and p_{branch} , calculated as the difference between the mean score across multiple simulation trials and the mean score across multiple OCTA images. (c) The heat map of the global vascular orientation $\overline{\sin^2(\theta_{\text{model}} - \theta_{\text{OCTA}})}$ for various $v_{\text{astrocyte}}$ and $d_{\text{inhibitor}}$. (d) The heat map of the vascular verticality around FAZ $\overline{\sin^2(\phi_{\text{model}} - \phi_{\text{OCTA}})}$ for various $v_{\text{astrocyte}}$ and $d_{\text{inhibitor}}$.

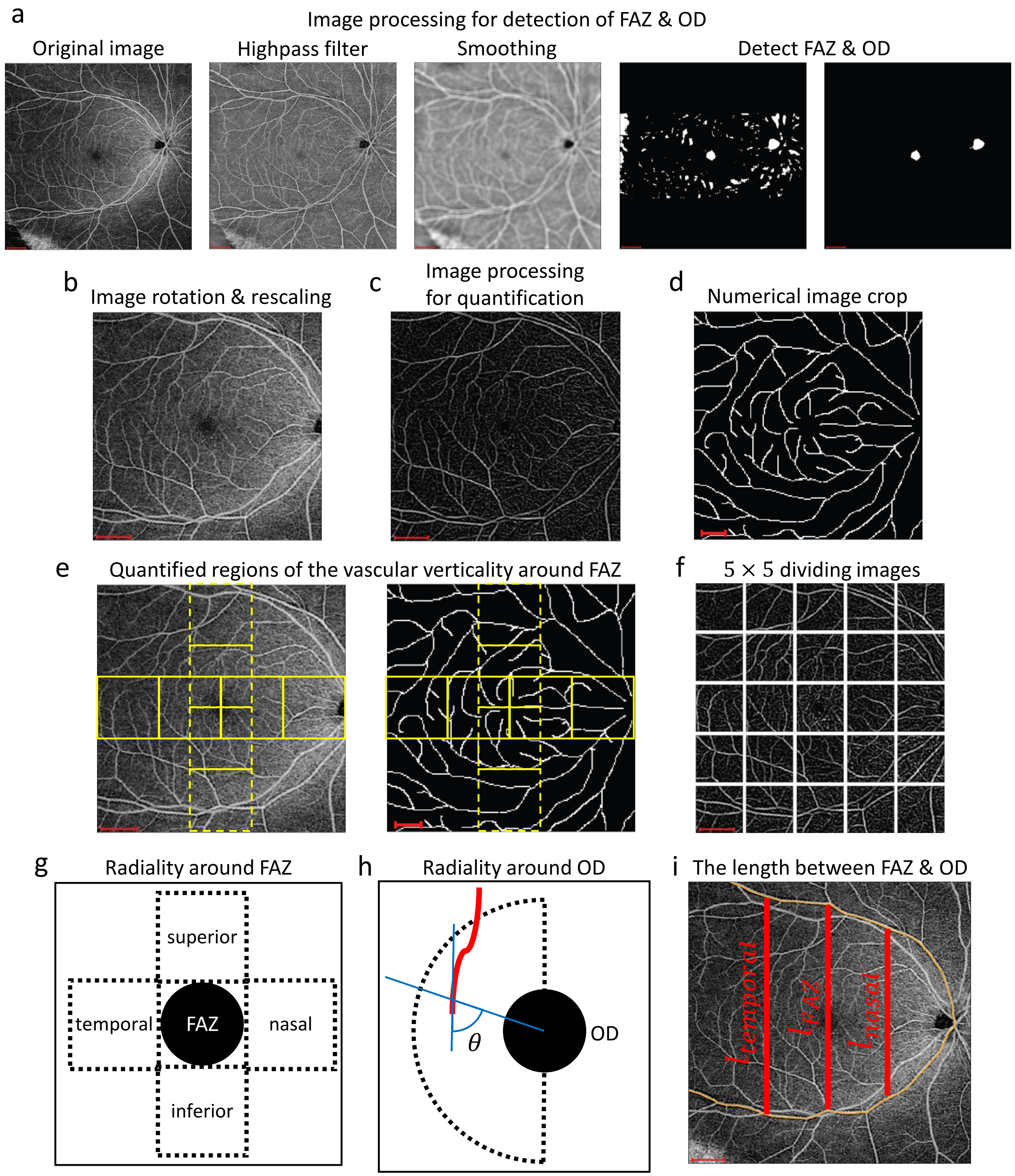

Figure S4. Image processing and quantification methods. (a) The flowchart for the detection of FAZ and OD in OCTA images. (b) A representative scaled OCTA image, which was rotated and cropped. (c) A representative processed OCTA image. (d) A representative scaled model result. (e) The areas defined for quantifying the vascular verticality around FAZ. Yellow solid boxes: temporal-nasal horizontal areas. Yellow dotted boxes: superior-inferior vertical areas. (f) A representative cropped 5×5 windows. (g) Four regions for quantifying the vascular radiality toward FAZ. (h) The schema of the quantification of the vascular radiality toward OD. (i) The schema illustrating the three quantified lengths l_{FAZ}, l_{nasal}, and l_{temporal}, defined to evaluate the characteristic features of the arcade vessels. Scale bars: 1 mm.

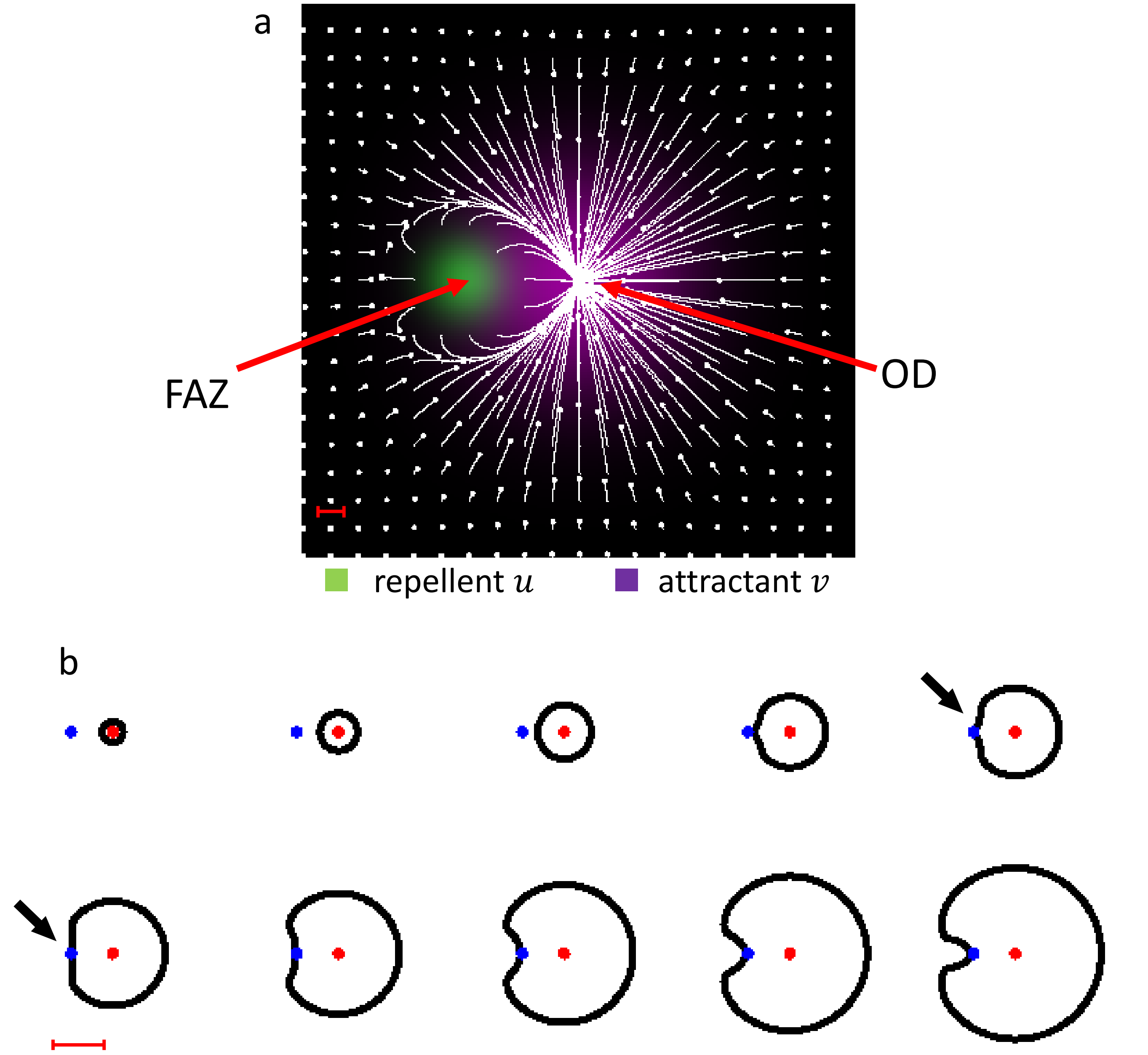

Figure S5. Astrocyte migration model based on retinal ganglion cell axons. (a) The vector field $\vec{V}_{\mathrm{axon}}$ in retinal ganglion cell axon development model. Light green distribution: axon repellent $u(x,y)$. Purple distribution: axon attractant $v(x,y)$. Scale bars: 1 mm. (b) Time-course of simulation results. Arrows: a protrusive structure. Red: OD. Blue: FAZ. Scale bars: 5 mm.

a

b

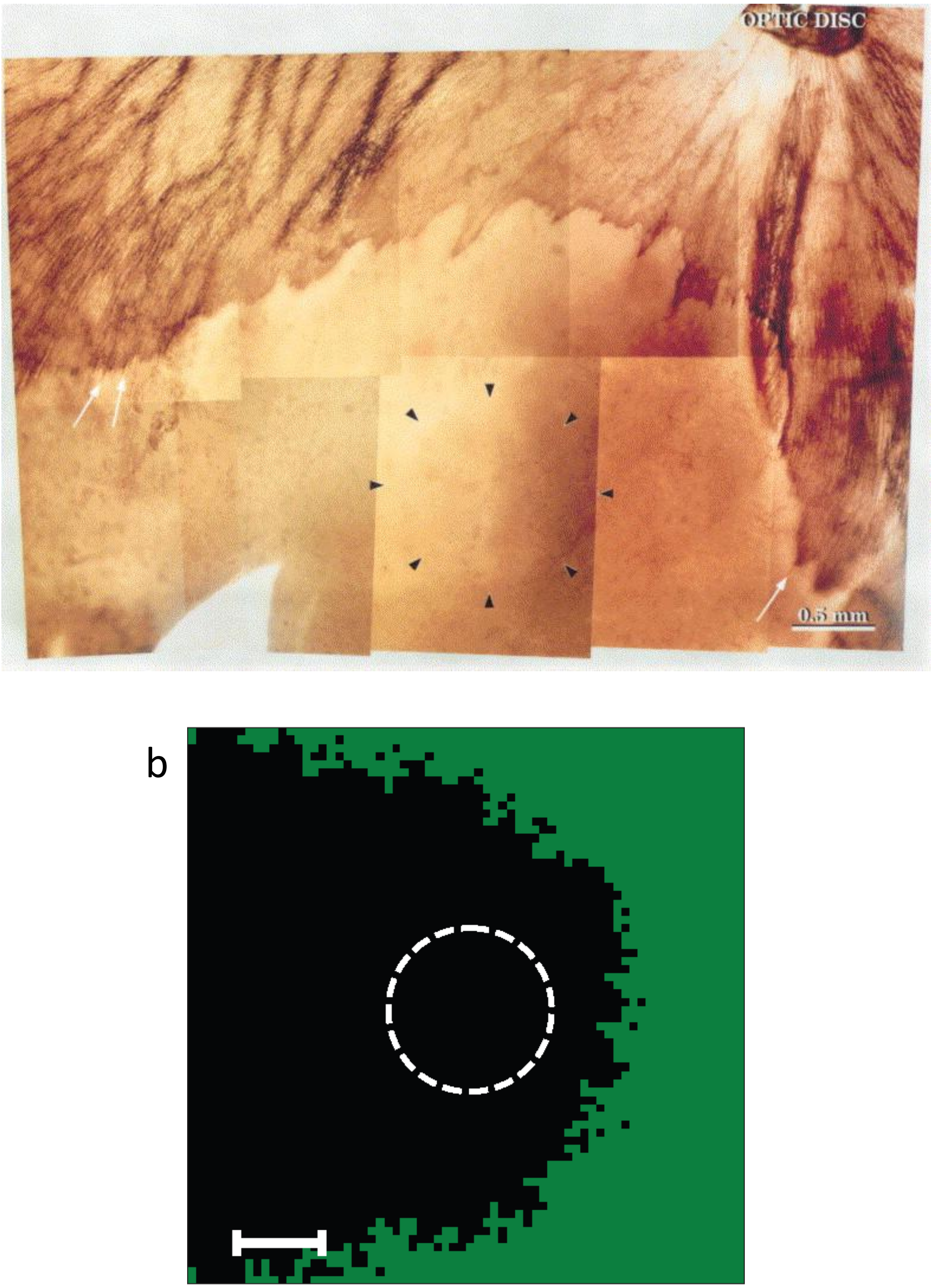

Figure S6. Jagged border of astrocyte distribution. (a) A photomontage showing the distribution of astrocytes, immuno-labeled with anti-glial fibrillary acidic protein (GFAP) and silver-intensified immunogold, in relation to the domed area (arrowheads) in a human foetus at 22 WG[57]. (b) Jagged border in our astrocyte dynamics model. Dotted circle: incipient FAZ. Scale bar: 0.5 mm.

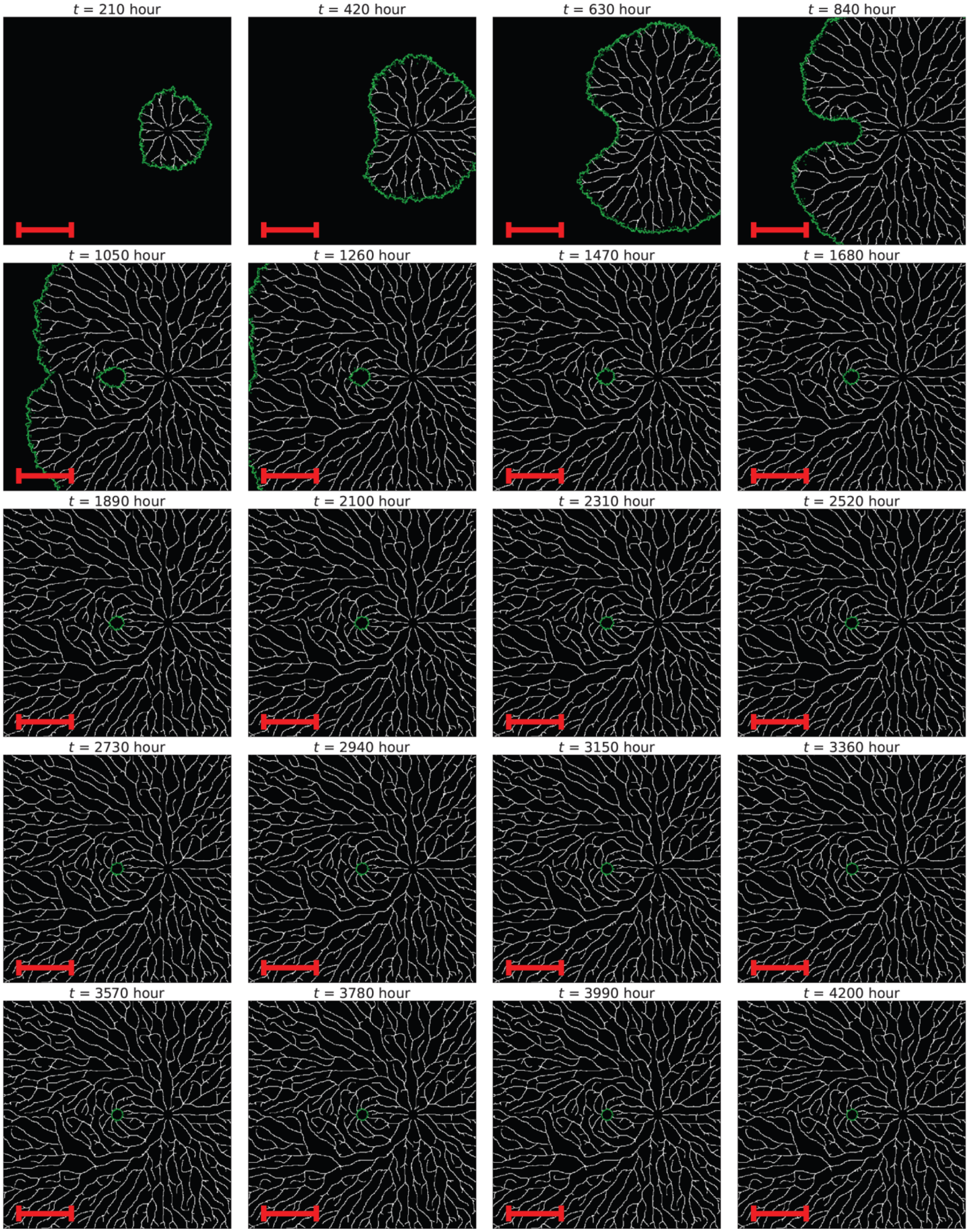

Figure S7. Long time course of our model. Scale bar: 5 mm.

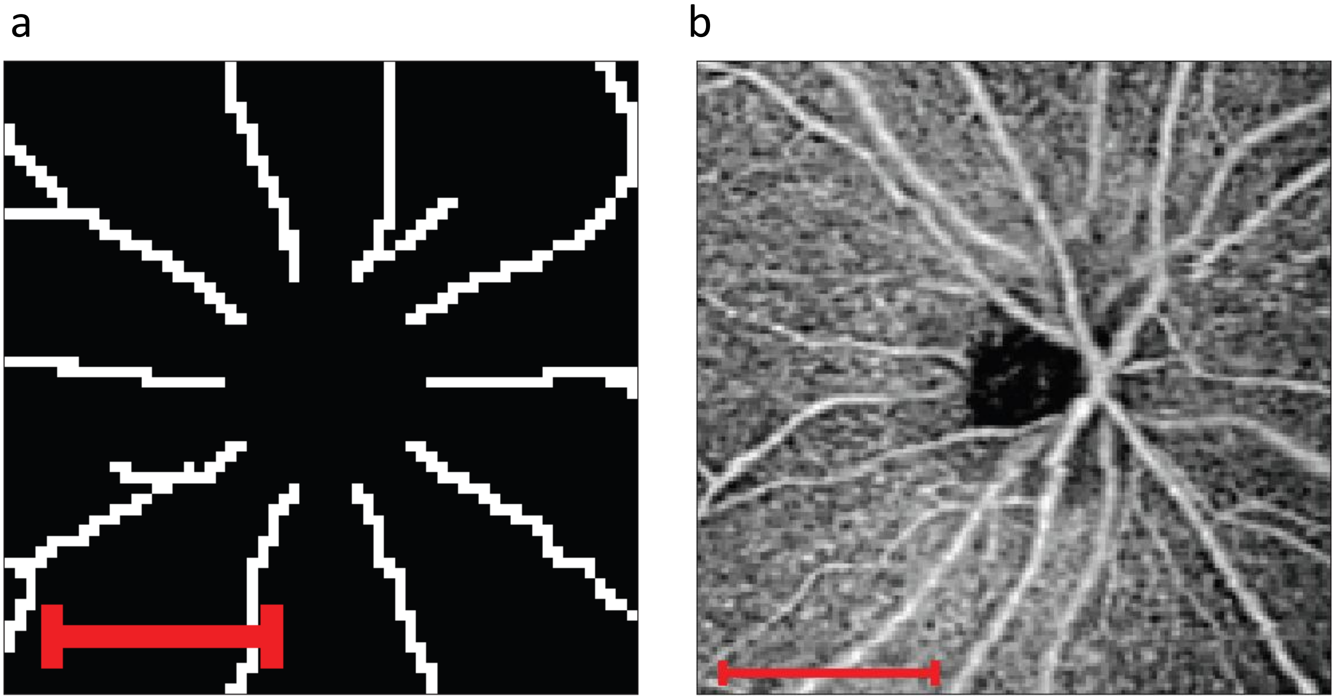

Figure S8. Close-up images around OD. (a) A numerical simulation. (b) An OCTA image, cropped from the corresponding original $12 \times 12mm$ image. Scale bars: 1 mm.

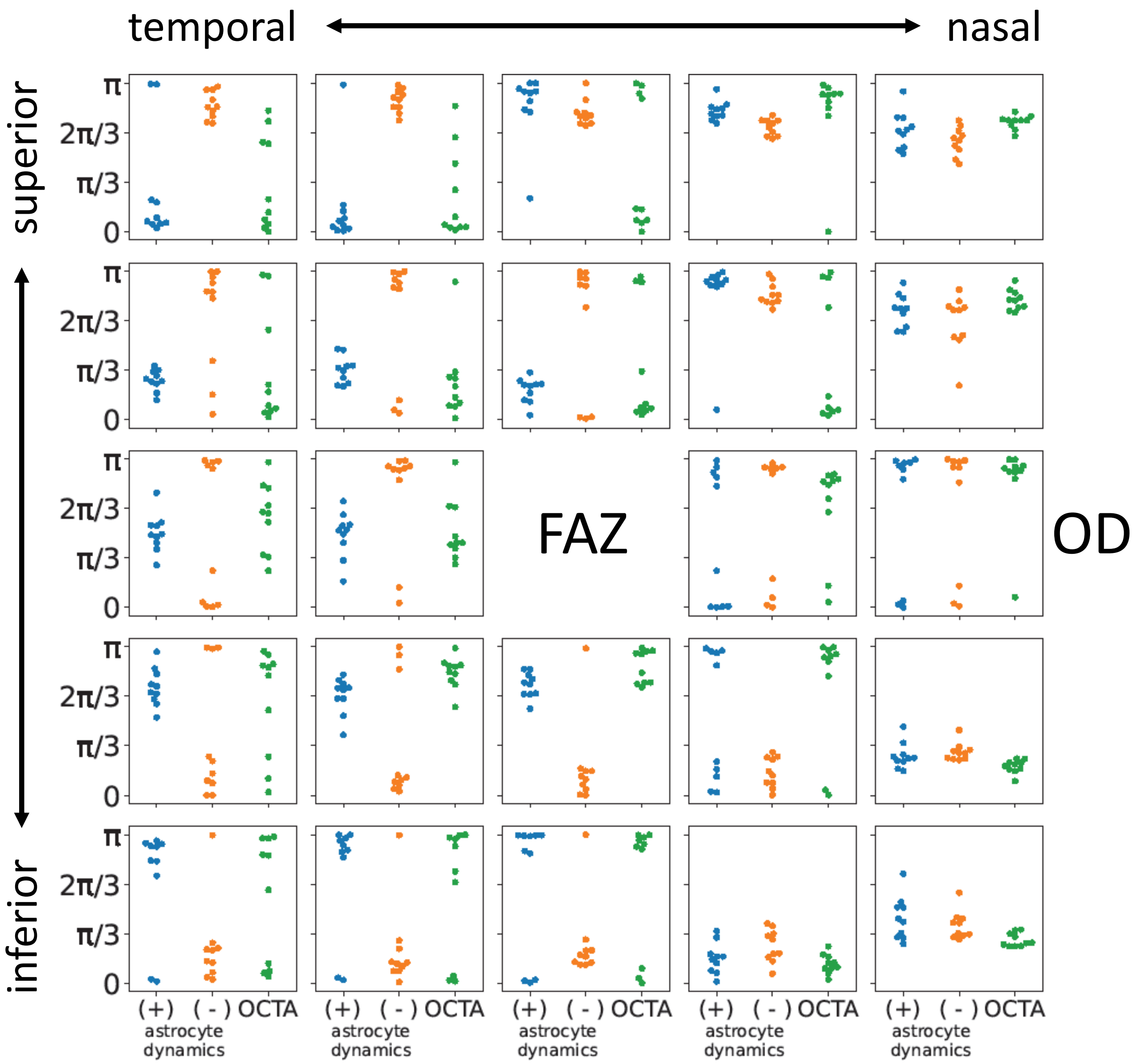

Figure S9. Quantitative comparison of the global vascular orientation for each area among the models with and without astrocyte dynamics (+/-) and OCTA images. mathematical models: $N = 20$, OCTA data: $N = 10$.

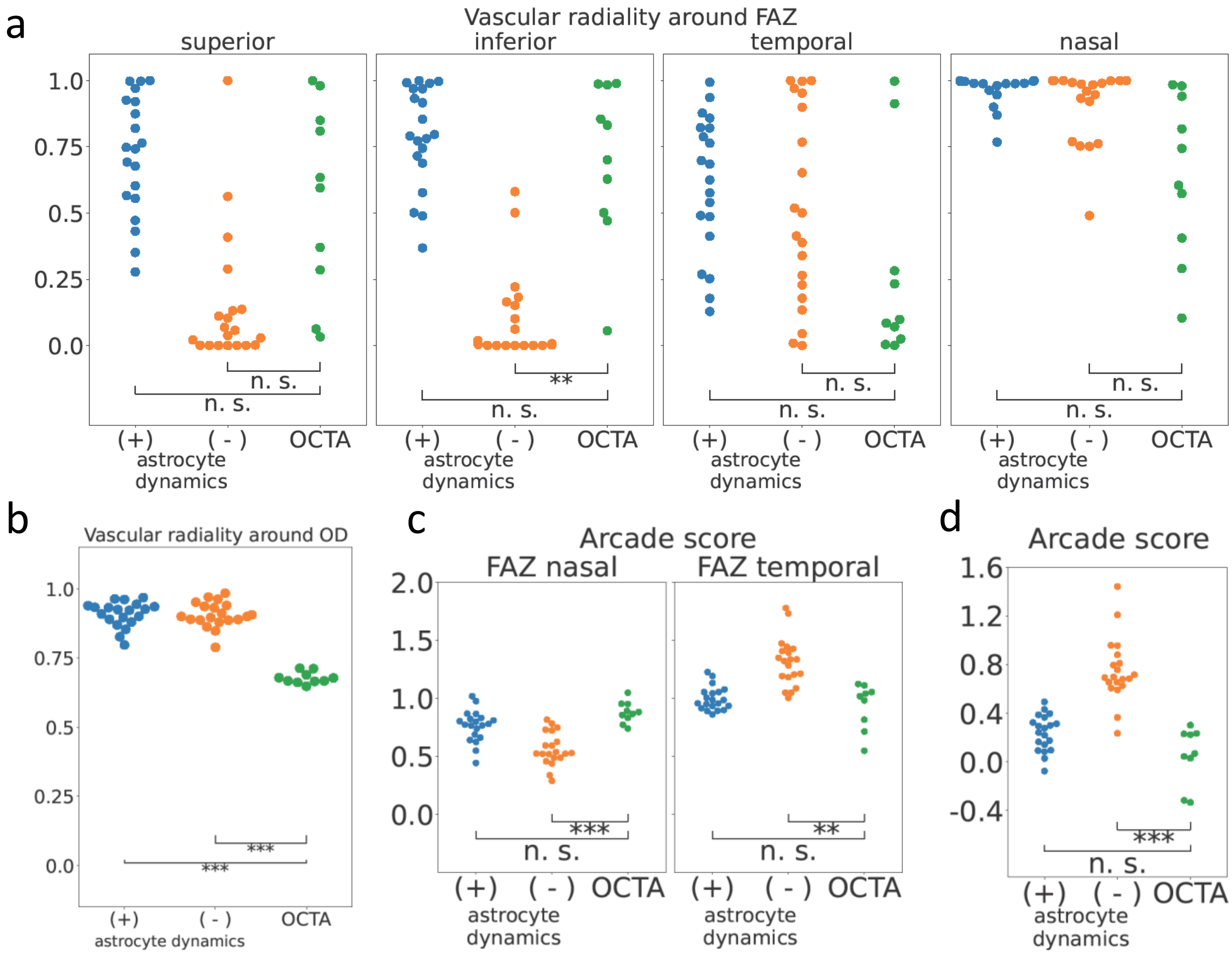

Figure S10. Quantitative comparison among the models with and without astrocyte dynamics (+/-) and OCTA images. (a) The vascular radiality toward FAZ from four regions. (b) The vascular radiality toward OD. (c) Arcade vessel score, calculated based on the distance between superior and inferior arcades. Left: $l_{\text{nasal}}/l_{\text{FAZ}}$. Right: $l_{\text{temporal}}/l_{\text{FAZ}}$. (d) The total scores based on (c), reflecting the arcade vessel curvature $(l_{\text{temporal}} - l_{\text{nasal}})/l_{\text{FAZ}}$. *: $p < 0.01$, **: $p < 0.001$, ***: $p < 0.001$ (Welch's t-test). Mathematical models: $N = 20$. OCTA data: $N = 9$ (temporal and total arcade scores) or $N = 10$ (others).

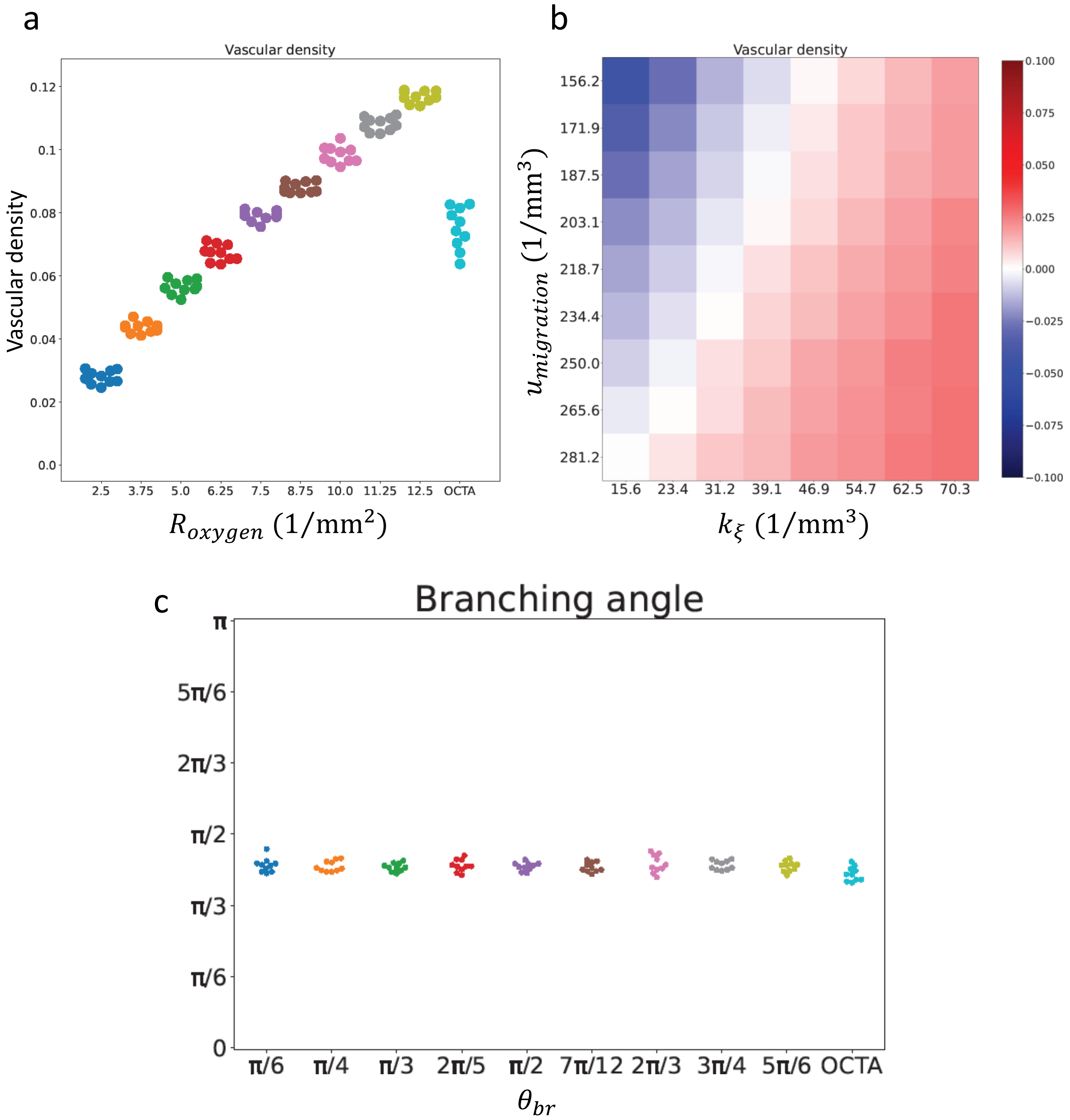

Figure S11. Sensitivity analyses of oxygen diffusivity, oxygen-dependent migration inhibition, and branching angle. (a) Vascular density for various R_{oxygen} and OCT data. (b) The heat map comparing OCTA data and numerical simulations for various $u_{\text{migration}}$ and k_ξ, calculated as the difference between the mean score across multiple simulation trials and the mean score across multiple OCTA images. (c) Branching angle for various θ_{br}. $N = 10$.

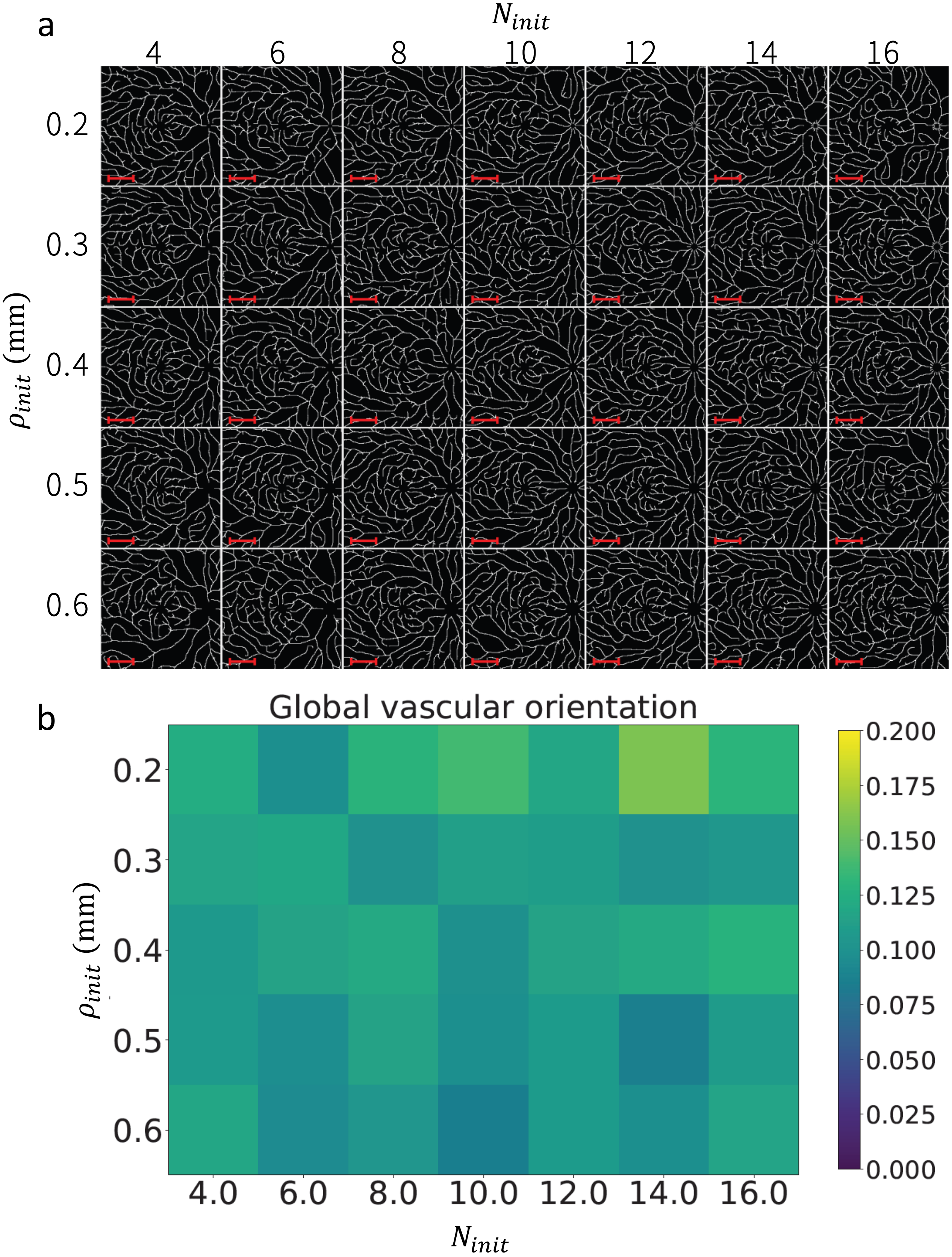

Figure S12. Sensitivity analyses of initial tip cell conditions. (a) Simulation results for various ρ_{init} and N_{init}. Scale bars: 2.5 mm. (b) The global vascular orientation for various ρ_{init} and N_{init}. $N = 10$.

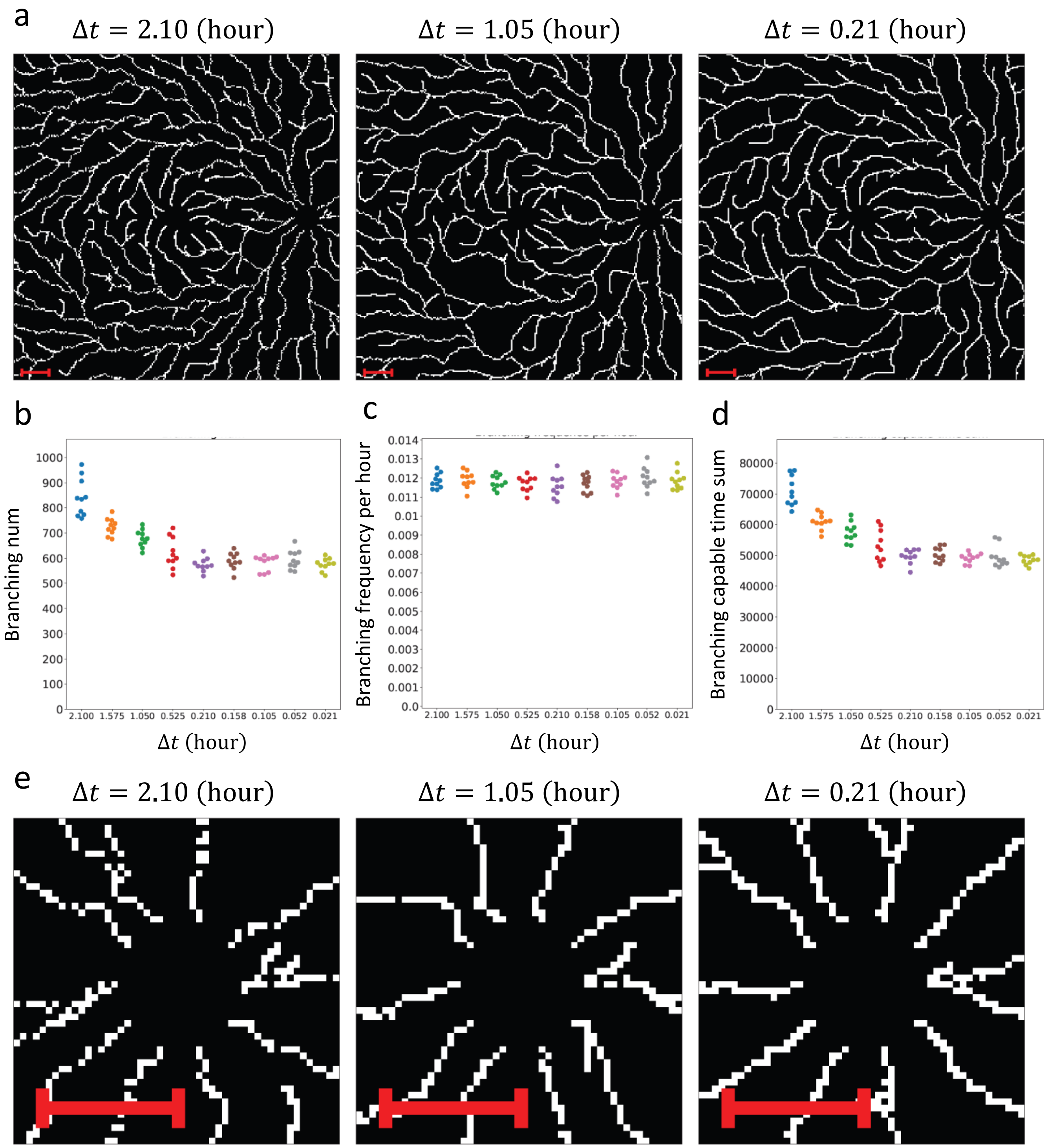

Figure S13. Sensitivity analyses of time discretization. (a) Simulation results for different Δt. (b) The number of branching events. (c) the branching frequency per time unit, limited to the period when tip cells were located in low-oxygen regions where branching is permitted. (d) The total duration during which tip cells were permitted to undergo branching. (e) Close-up of a simulation result around OD. Scale bars: 1 mm. $N = 10$.

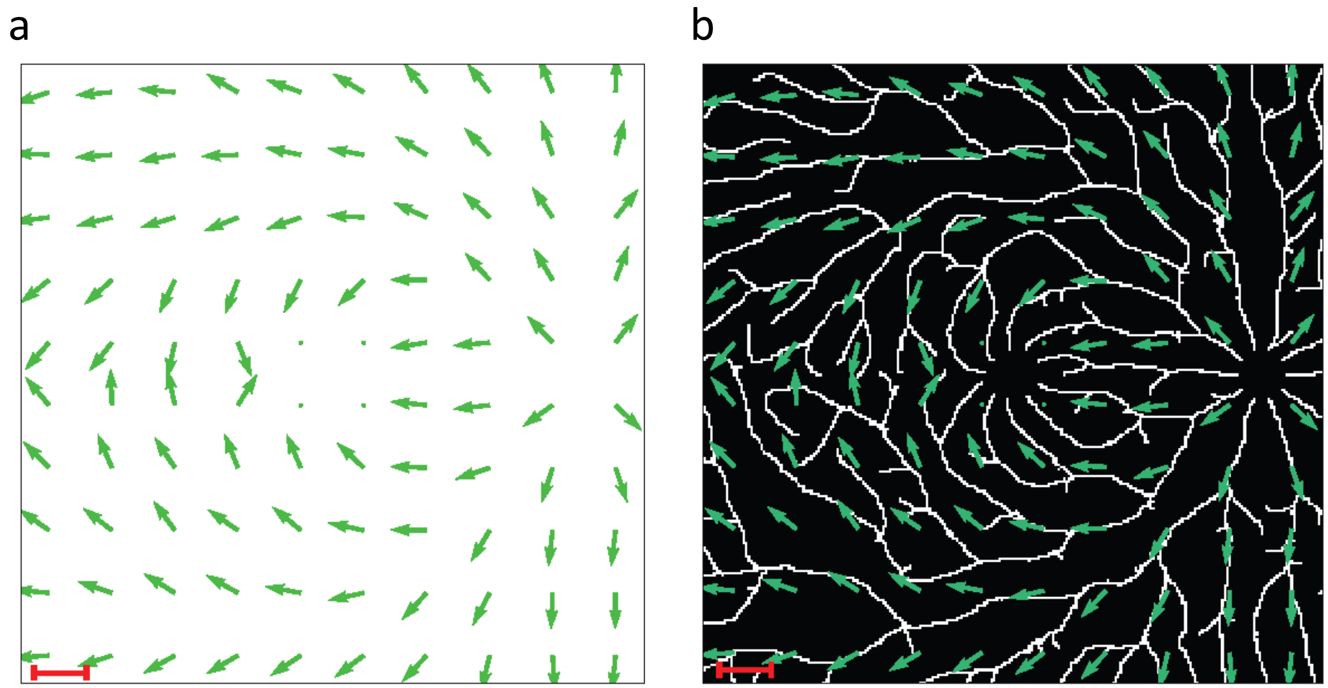

Figure S14. Astrocyte expansion velocity map. (a) A vector field of astrocyte expansion velocity in a numerical simulation. (b) An overlapping map of the astrocyte expansion velocity field and a vascular pattern in our model. Scale bars: 1 mm.

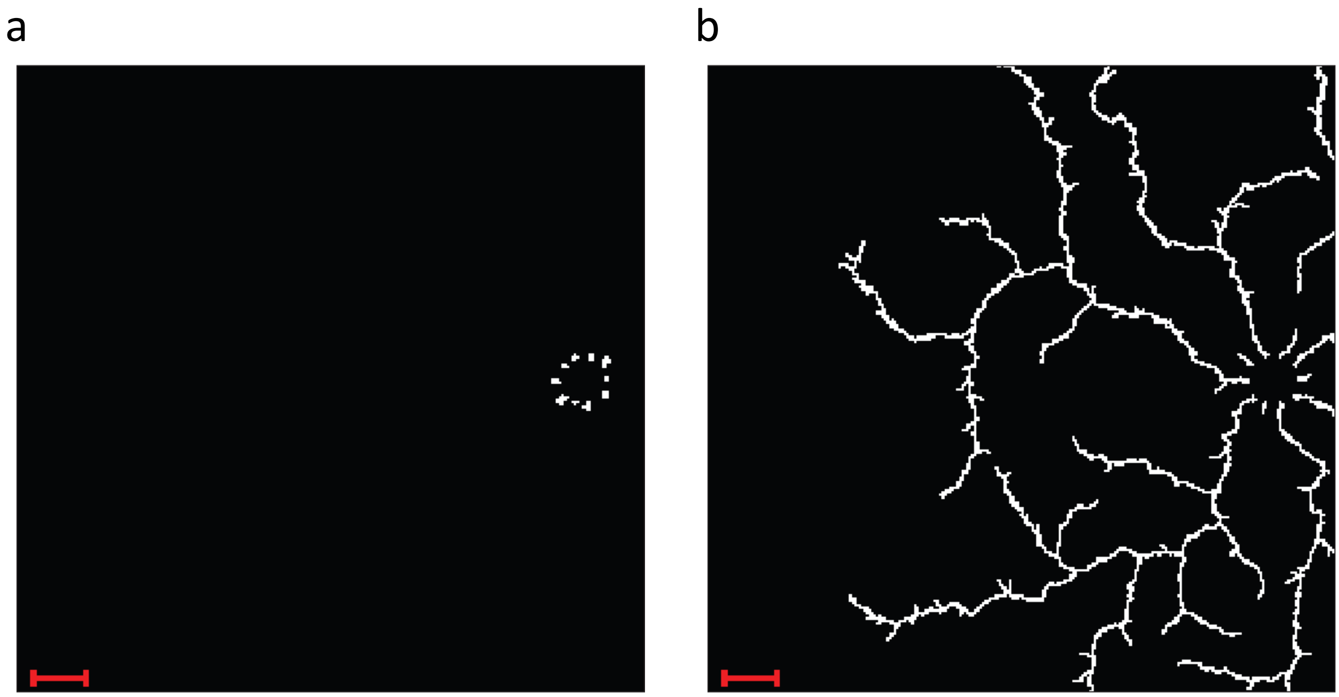

Figure S15. The numerical simulation results without chemotaxis (a, $\beta = 0$ (mm^5/hour)) and with weak chemotactic effects (b, $\beta = 2.682 \times 10^{-6}$ (mm^5/hour)). The magnifications and time points were the same as those in the top panels of Fig.4a. Scale bar: 1 mm.

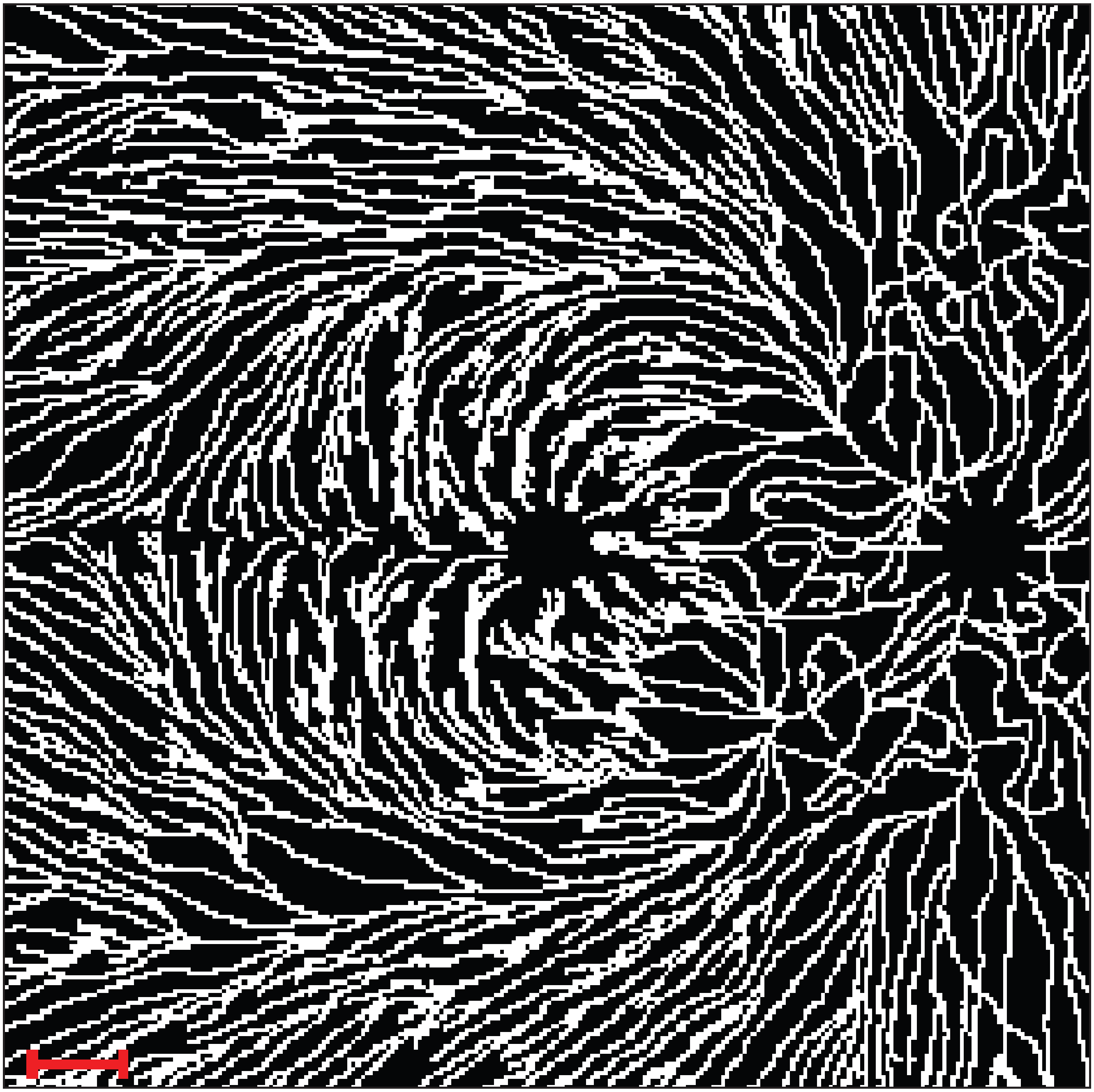

Figure S16. The numerical simulation results with the high u_{branch}. Scale bar: 1 mm.
