# Supplementary material for: Mathematical Modeling of Human Retinal Vascular Pattern Around the Foveal Avascular Zone
Source: Transl Vis Sci Technol. 2026 Mar 2;15(3):1. doi: 10.1167/tvst.15.3.1 (PMC12967130; doi:10.1167/tvst.15.3.1)
Supplement: Supplement 4 [file tvst-15-3-1_s004.pdf]

Supporting information

S1 Assessment of OCTA image distortion caused by the projection of spherical surface into the plane

The retinal vascular pattern around FAZ may be influenced by the projection of the vascular structure on the spherical retinal surface into a plane. Therefore, we assessed the image distortion of retinal vasculature induced by this projection. A retina was assumed as a hemispherical surface I of radius r ($z \geq 0$), centered at the origin O (Supplementary Fig. S1a). To consider how radially elongated vessels from the optic disc are projected onto the plane, we also defined a point P on the surface inclined $\theta_0 = \frac{\pi}{12}$ from the apex as optic disc[17] and an arbitrary point Q on the great circle of the hemisphere (Supplementary Fig. S1a):

$$\overrightarrow{OP} = (r\sin\frac{\pi}{12}, 0, r\cos\frac{\pi}{12}), \tag{25}$$

$$\overrightarrow{OQ} = (r\cos\theta, r\sin\theta, 0) \qquad (0 \leq \theta < \pi). \tag{26}$$

We then considered the plane H spanned by $\overrightarrow{OP}$ and $\overrightarrow{OQ}$ and its intersecting semicircle with the hemisphere (Supplementary Fig. S1a,b). Any point S on the semicircle is described as

$$\overrightarrow{OS} = \overrightarrow{OQ}\cos\psi + \overrightarrow{OR}\sin\psi, \tag{27}$$

where $0 \leq \psi \leq \pi$ and $\overrightarrow{OQ} \perp \overrightarrow{OR}$.

$\overrightarrow{OR}$ was calculated as follows. $\overrightarrow{OP}$, $\overrightarrow{OQ}$, and $\phi := \angle POQ$ satisfy the following relationship,

$$\overrightarrow{OP} \cdot \overrightarrow{OQ} = \left|\overrightarrow{OP}\right| \left|\overrightarrow{OQ}\right| \cos\phi = r^2 \sin\frac{\pi}{12}\cos\theta. \tag{28}$$

Hence,

$$\cos\phi = \sin\frac{\pi}{12}\cos\theta, \tag{29}$$

$$\sin\phi = \sqrt{1 - \sin^2\frac{\pi}{12}\cos^2\theta}. \tag{30}$$

Note that $\sin\phi \geq 0$. Then, we considered the vector $\overrightarrow{OT} = a\overrightarrow{OP}$ $(a > 0)$ that satisfies $\overrightarrow{RT} || \overrightarrow{OQ}$. We obtain

$$r = ar\sin\phi, \tag{31}$$

$$\overrightarrow{OR} = \overrightarrow{OT} - a\cos\phi\overrightarrow{OQ} = a\overrightarrow{OP} - a\cos\phi\overrightarrow{OQ}. \tag{32}$$

Therefore,

$$\overrightarrow{OR} = \frac{r}{\sqrt{1-\sin^2\frac{\pi}{12}\cos^2\theta}}\left(\sin\frac{\pi}{12}\sin^2\theta, -\sin\frac{\pi}{12}\sin\theta\cos\theta, \cos\frac{\pi}{12}\right). \tag{33}$$

By applying Eq. (26) and Eq. (33) to Eq. (27), we calculated the trajectory of point S in the range $0 \leq \psi < \pi$ and visualized the projection of the semicircle onto the plane $z = 0$ (Supplementary Fig. S1c). We also showed its enlarged image, corresponding to the area shown in the OCTA images, in Supplementary Fig. S1d. These results indicated that the distortion due to the projection alone cannot explain the vascular pattern around FAZ. Therefore, we investigated the biological mechanism for human retinal vascular pattern formation.

S2 Effect of retinal ganglion cell axons on astrocyte migration

Astrocytes follow retinal ganglion cells (RGCs) axons during their expansion in retinas[60]. Before astrocytes invade the retina from the optic disc, RGC axons have already been distributed in the retina[71]. Therefore, we speculated that RGC axons may have some influence on the astrocyte migration pattern. In the human retina, RGC axons radiate out from the fovea and project to terminate at the optic disc[20,72]. During retinal development, FGF8 is highly expressed in the high-acuity area in chick embryos, which corresponds to the fovea in primates[59]. It is also reported that FGF8-soaked beads increase Semaphorin 3F expression in rat brains and that Semaphorin 3F inhibits the axonal growth of midbrain dopaminergic neurons[73]. On the other hand, Sonic Hedgehog, expressed around the optic disc in chick embryos, is critical for the centrally directed projection of RGC axons[74]. Based on these reports, we assumed a factor that repels RGCs axons from the fovea ($u(x,y)$) and another factor that attracts RGCs axons to the optic disc ($v(x,y)$):

$$u(x,y) = w_{\text{repel}} \exp\left(-\frac{(x-x_{\text{fovea}})^2 + (y-y_{\text{fovea}})^2}{\rho_{\text{repel}}^2}\right), \tag{34}$$

$$v(x,y) = w_{\text{attract}} \exp\left(-\frac{(x-x_{\text{OD}})^2 + (y-y_{\text{OD}})^2}{\rho_{\text{attract}}^2}\right), \tag{35}$$

where ρ_{attract}, ρ_{repel}, w_{attract}, and w_{repel} were the diffusion length of the attractant, the diffusion length of the repellent, the coefficient of the attractant, and the coefficient of the repellent respectively. We modeled that the axon elongation $\vec{V}_{\text{axon}}$ from an RGC body is governed by the combination of chemoattraction towards the optic disc and chemorepulsion from the fovea:

$$\vec{V}_{\text{axon}} = c_{\text{axon}}\left(-\alpha\nabla u + \beta\nabla v\right). \tag{36}$$

This model showed a similar pattern to that of RGC axons (Supplementary Fig. S5a), which had a radial pattern avoiding the foveal center[20].

Next, we investigated the astrocyte expansion time course, assuming that they followed the RGC axon pattern. Astrocytes

were assumed to spread with constant velocity $c_{\text{astrocyte}}$ from the optic disc. We defined a vector field $\vec{V}_{\text{astrocyte}}$ as follows:

$$\vec{V}_{\text{astrocyte}} := -c_{\text{astrocyte}} \frac{\vec{V}_{\text{axon}}}{|\vec{V}_{\text{axon}}|}. \tag{37}$$

Assuming that astrocytes spread along this vector field, we computed the displacement of the astrocyte boundary. A protrusive structure between the optic disc and the fovea appeared in the middle of the time course (Supplementary Fig. S5b arrows). This protrusion resulted from the difference in length from the optic disc to the fovea along both the straight and curved paths. Such astrocyte structure was not reported in the human developmental retina. We did not deny that RGCs contribute to the astrocyte development, as we tested a simple assumption which included them. However, it is unlikely that the developmental pattern of astrocytes would be established solely based on the effects of RGCs. These results also support our assumption about the presence of an astrocyte inhibitor in the model.

In this section, numerical simulations of the model were implemented by Mathematica (Wolfram Research), which provides an "Interpolation" function suitable for calculating the displacement of the astrocyte border.

S3 Calculation of the vector field of the astrocyte expansion

We defined $\Gamma(\vec{r})$ as the sum of the astrocyte distribution $\gamma(\vec{r})$ for timepoints and considered its spatial gradient $\nabla\Gamma(\vec{r}) = (\nabla\Gamma_x, \nabla\Gamma_y)$. The vector field denoting the astrocyte expansion velocity is then expressed as $\left(-\frac{1}{\nabla\Gamma_x}, -\frac{1}{\nabla\Gamma_y}\right)$.

S4 Parameter selection in our model

To select the optimal parameters for our model, we first analyzed how varying the parameters α and β affected the vessel tortuosity of our model. We computed the vessel tortuosity in the range of $3.81 \times 10^{-3} \leq \alpha \leq 1.333 \times 10^{-2}$ (mm/hour) and $9.8 \times 10^{-6} \leq \beta \leq 2.93 \times 10^{-5}$ (mm^5/hour) (Supplementary Fig. S3a). We selected $\alpha = 1.238 \times 10^{-3}$ (mm/hour) and $\beta = 2.682 \times 10^{-5}$ (mm^5/hour).

Next, to establish the parameters u_{branch} and p_{branch}, which are related to the vascular density, we explored their optimal values between $156.2 \leq u_{\text{branch}} \leq 296.9$ (1/mm^3) and $4.8 \times 10^{-3} \leq p_{\text{branch}} \leq 5.95 \times 10^{-2}$ (1/hour) (Supplementary Fig. S3b). We found $u_{\text{branch}} = 234.4$ (1/mm^3) and $p_{\text{branch}} = 1.19 \times 10^{-2}$ (1/hour).

We also selected the suitable values for $v_{\text{astrocyte}}$ and $d_{\text{inhibitor}}$ within the range of $4.76 \times 10^{-3} \leq v_{\text{astrocyte}} \leq 6.09 \times 10^{-3}$ (mm/hour) and $1.2 \leq d_{\text{inhibitor}} \leq 4.4$ (mm). These parameters highly influenced the vascular pattern, especially around FAZ (Supplementary Fig. S3c,d). For our model, we applied $v_{\text{astrocyte}} = 5.90 \times 10^{-3}$ (mm/hour) and $d_{\text{inhibitor}} = 2.4$ (mm), which resulted in good agreement with the OCTA images regarding both the global vascular orientation and the vascular verticality around FAZ.

S5 Parameter variation

S5.1 Oxygen diffusivity

We also tested the relationship between the cell density and the oxygen diffusion length, which was controlled by q_{oxygen} and D_{oxygen} in Eq. (5). We varied $R_{\text{oxygen}} := \frac{q_{\text{oxygen}}}{D_{\text{oxygen}}}$ and observed its effect on the cell density, as only R_{oxygen} affected the oxygen kernel k_{oxygen}. These results showed a linear increase of the vascular density depending on R_{oxygen} (Supplementary Fig. S11a).

S5.2 Oxygen-dependent tip cell behavior

We explored how the parameters in Eq. (10) affected the cell density. In this exploration, we did not assume that the oxygen threshold for decelerating the tip cell migration was equal to u_{branch}. Therefore, we investigated the cell density in the range of $15.6 \leq k_\xi \leq 70.3$ (1/mm^3) and $156.2 \leq u_{\text{migration}} \leq 281.2$ (1/mm^3) on the following term:

$$\xi(\vec{r}) = \begin{cases} 1 & u(x,y,t) < u_{\text{migration}}, \\ \exp\left(-\frac{u(x,y,t)-u_{\text{migration}}}{k_\xi}\right) & u(x,y,t) \geq u_{\text{migration}}. \end{cases} \tag{38}$$

The vascular density increased based on both parameters, which reflected that the vessels in our model became longer (Supplementary Fig. S11b).

S5.3 Branching angle

We examined the relationship between the set parameter angle θ_{br} and the actual angle of the branches in our model and OCTA images. However, the set branching angle θ_{br} did not affect the apparent branch angles (Supplementary Fig. S11c). The other parameters (ρ_{FAZ}, k_i, $\rho_{\text{astrocyte}}$, l_{vessel}, and ρ_{retina}) were selected based on visual similarity determined between the actual and simulated patterns, as evaluated by the ophthalmologists.

S5.4 Initial conditions

We investigated how the numerical simulation results vary depending on the different initial conditions, the number of initial tip cells N_{init}, and the initial radius of tip cell position ρ_{init}. Few tip cells stop around OD with larger N_{init} or smaller ρ_{init}, which would be explained by excessive oxygen around the initial position (Supplementary Fig. S12a). However, the global vascular orientation did not differ among various initial conditions qualitatively and quantitatively (Supplementary Fig. S12a,b).

S5.5 Time step

We also examined the effect of different time steps for discretization. As Δt decreased, the branching frequency also decreased in the numerical simulations (Supplementary Fig. S13a). Although the numbers of branching events per time unit decreased under small Δt (Supplementary Fig. S13b), the branching frequency per time unit, limited to the period when tip cells were located in low-oxygen regions where branching is permitted, remained consistent with the predefined frequency p_{branch} (Supplementary Fig. S13c). Therefore, we assessed the total duration during which tip cells were permitted to undergo branching. The duration became longer with larger Δt (Supplementary Fig. S13d). These variations of branching properties reached a plateau when

Δt was sufficiently small (Supplementary Fig. S13b,d). In the numerical simulations, we found that the vessels simulated with smaller Δt occupied more lattice pixels than those with larger Δt, which can be explained by the shorter migration of tip cells in one numerical step (Supplementary Fig. S13e). We considered that these vascular changes in our model led to increased oxygen concentration at the tip cell locations, and then, the decrease in branching events with small Δt. Therefore, we employed $\Delta t = 0.21$ (hour) in the numerical simulations, which was the maximum value within the range where these branching properties remained nearly unchanged.

S6 Supporting tables

Parameter	Description	Value
α	coefficient of random cell migration	1.238×10^{-3} [mm/hour]
β	coefficient of chemotaxis	2.682×10^{-5} [mm^5/hour]
u_0	coefficient of the oxygen supply	15.625 [1/mm^3]
q_{oxygen}	consumption rate of oxygen	4.762×10^{-4} [1/hour]
D_{oxygen}	diffusion coefficient of oxygen	7.169×10^{-5} [mm^2/hour]
R_{oxygen}	ratio of the oxygen consumption rate to its diffusion coefficient	6.25 [1/mm^2]
p_{branch}	branching probability	1.19×10^{-2} [1/hour]
u_{branch}	oxygen concentration threshold for branching	234.4 [1/mm^3]
$u_{\text{migration}}$	oxygen concentration threshold for tip cell migration	234.4 [1/mm^3]
k_ξ	coefficient of oxygen inhibition for tip cell migration	31.25 [1/mm^3]
ρ_{init}	the radius of initial tip cell positions	0.4 [mm]
θ_{br}	branching angle	$\frac{2}{5}\pi$
d_{OD}	distance between the optic disc and the fovea	4 [mm]
ρ_{FAZ}	FAZ radius	0.4 [mm]
$v_{\text{astrocyte}}$	astrocyte spreading velocity without its inhibitor	5.90×10^{-3} [mm/hour]
k_i	coefficient of the astrocyte inhibitory effects	6.4×10^{-3} [mm^3]
$d_{\text{inhibitor}}$	diffusive length of the astrocyte inhibitor	2.4 [mm]
$\rho_{\text{astrocyte}}$	initial radius of the astrocyte distribution	0.8 [mm]
N_{init}	initial tip cell number	10
l_{vessel}	diameter of the vascular vessels	0.04 [mm]
ρ_{retina}	simulation domain radius (retina)	20 [mm]
$\rho_{\text{endo,inh}}$	the diffusion length of the endothelial inhibitory molecule	0.4 [mm]
ρ_{VEGF}	the diffusion length of the VEGF	0.4 [mm]
Δx	lattice size	0.04 [mm]
Δt	time step	0.21 [hour]

Table 1. Model parameter values used in this study.

S7 Supporting videos

Supplementary Video 1. The retinal astrocyte expansion model. Left: the whole retina. Scale bar: 5 mm. Right: the region around the fovea. Scale bar: 1 mm.

Supplementary Video 2. The combined model of angiogenesis and astrocyte expansion. The green line is the border of astrocyte distribution. Left: the whole retina. Scale bar: 5 mm. Right: the region around the fovea. Scale bar: 1 mm.
